# CopyCatchers are versatile active genetic elements that detect and quantify inter-homolog somatic gene conversion

**DOI:** 10.1038/s41467-021-22927-1

**Published:** 2021-05-11

**Authors:** Zhiqian Li, Nimi Marcel, Sushil Devkota, Ankush Auradkar, Stephen M. Hedrick, Valentino M. Gantz, Ethan Bier

**Affiliations:** 1 Section of Cell and Developmental Biology, University of California San Diego, La Jolla, CA USA; 2 Section of Molecular Biology, University of California San Diego, La Jolla, CA USA; 3 Department of Cellular and Molecular Medicine, University of California San Diego, La Jolla, CA USA; 4 Tata Institute for Genetics and Society-UCSD, La Jolla, CA USA

**Keywords:** Synthetic biology, CRISPR-Cas9 genome editing

## Abstract

CRISPR-based active genetic elements, or gene-drives, copied via homology-directed repair (HDR) in the germline, are transmitted to progeny at super-Mendelian frequencies. Active genetic elements also can generate widespread somatic mutations, but the genetic basis for such phenotypes remains uncertain. It is generally assumed that such somatic mutations are generated by non-homologous end-joining (NHEJ), the predominant double stranded break repair pathway active in somatic cells. Here, we develop CopyCatcher systems in *Drosophila* to detect and quantify somatic gene conversion (SGC) events. CopyCatchers inserted into two independent genetic loci reveal unexpectedly high rates of SGC in the *Drosophila* eye and thoracic epidermis. Focused RNAi-based genetic screens identify several unanticipated loci altering SGC efficiency, one of which (*c-MYC*), when downregulated, promotes SGC mediated by both plasmid and homologous chromosome-templates in human HEK293T cells. Collectively, these studies suggest that CopyCatchers can serve as effective discovery platforms to inform potential gene therapy strategies.

## Abstract (web summary)

CRISPR-based genetic elements can copy themselves across host genomes. Here the authors introduce CopyCatcher, a gene-drive related system for detecting and quantifying somatic gene conversion events.

## Introduction

CRISPR-based active genetic elements are self-propagating cassettes carrying gRNAs (±Cas9 or associated cargos) that cut the genome at the location where those elements are inserted^1^. Active genetic elements, particularly gene-drives (carrying linked gRNA and Cas9 transgenes), offer great potential for population modification or suppression by repairing double-strand breaks (DSBs) through homology-directed repair (HDR) in germline cells to disseminate beneficial genetic cargo throughout an insect population or to decrease (suppress) the population size^1–3^. Alternatively, DSBs can be repaired through the error-prone non-homologous end-joining (NHEJ) pathway, by ligating the two broken DNA ends together or creating indels at the cut site when challenged by repeated Cas9 cleavage. NHEJ is active throughout the cell cycle, rather than being confined to late S and G_2_ phases as is HDR. NHEJ is thus considered to be the primary DSB repair pathway in somatic cells under normal circumstances^4–10^.

While CRISPR-based gene-drive elements can be highly efficient in copying themselves in germline cell lineages, the general view has been that CRISPR induced DSB in somatic cells are repaired predominantly by the NHEJ pathway^1,2,7,11–15^ and that if cleavage-resistant mutations generated by imprecise NHEJ repair arise during early embryonic stages (prior to allocation of the germline), they reduce subsequent drive efficiency in the germline^2,16–23^. This interpretation of NHEJ alleles limiting gene-drive performance is also consistent with the lower efficiency of HDR relative to NHEJ typically observed in cultured mammalian cells^7,24^.

When engaging the HDR pathway, somatic cells maintain genome integrity by employing identical sister chromatids as DSB repair templates. This post-replicative and restorative function of HDR in somatic cells contrasts with its role in the germline where meiotic factors promote DSB-dependent recombination between homologous chromosomes^25,26^. Also, dysfunctional engagement of HDR in which the homologous chromosome rather than the sister chromatid serves as the repair template results in loss-of-heterozygosity (LOH) phenotypes that can lead to oncogenic outcomes and developmental defects^27,28^. Collectively, these considerations support the current hypothesis that somatic cells employ either NHEJ or sister chromatid-based HDR as the predominant DSB repair strategies, providing a ready explanation for the relatively low rates of precise gene editing typically achieved when using exogenously provided DNA templates.

Although DSB repair mechanisms differ between germline and somatic cells in several important respects, a set of core factors play essential roles in both repair processes including: Ku70/Ku80 heterodimers^29^, RAD51^30^, MRE11^29,31^, CtIP^32–34^ and 53BP1^35,36^. How other cellular processes, including chromosome pairing and remodeling, might influence DSB repair, particularly in somatic cells, remains largely unknown.

In the current study, we develop CRISPR-based active genetic CopyCatcher systems to detect and quantify genetic somatic gene conversion (SGC) events in vivo in *Drosophila*. CopyCatchers reveal unexpectedly high rates of SGC in *Drosophila* and can be employed as versatile tools for identifying genetic components required for the SGC process. Homolog-templated SGC can also take place in mammalian cells, and loci impacting the rates of such repair (e.g., *c-MYC*) function in a conserved fashion in this process. Collectively, these results suggest that CopyCatchers offer efficient systems for tracking and dissecting homolog-based copying mechanisms in somatic cells and offer a potential avenue for pursuing precise human gene therapy.

## Results

### The architecture of active genetic CopyCatcher systems

**Fig. 1:**
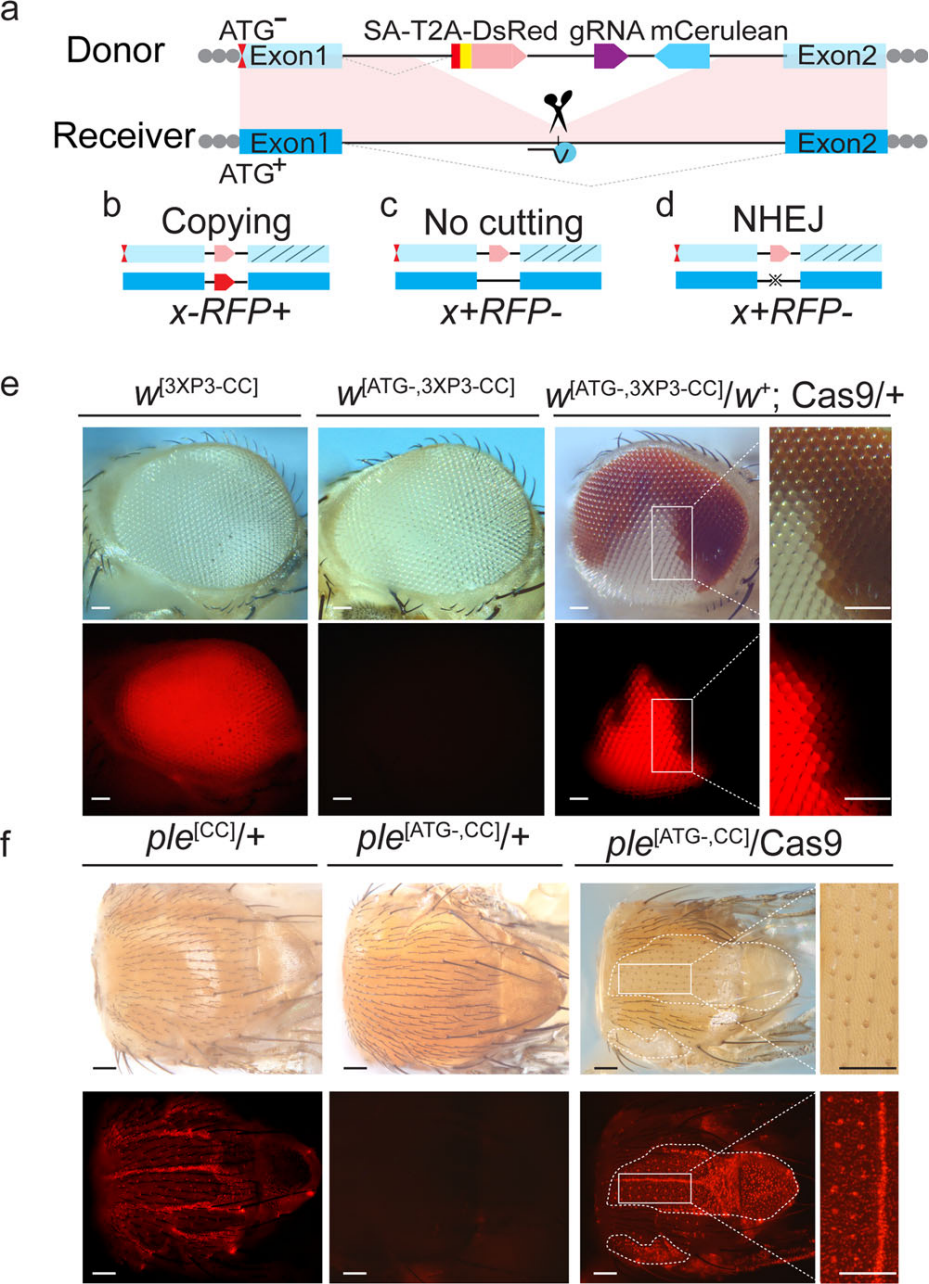
Schematic representation of CopyCatcher system. **a** Scheme depicting a generic CopyCatcher element. Light blue boxes: exons of a targeted gene on the donor chromosome; dark blue boxes: exons on the receiver chromosome; black lines: genomic DNA; red box: splice acceptor site (SA); yellow box: T2A self-cleavage peptide; light red arrow: *DsRed* reporter; dark purple arrow: gRNA; light blue arrow: selection marker *mCerulean*; red hourglass marks: point mutations at or near initiator ATG codons of endogenous genes; short black line: Cas9/gRNA cleavage site; light blue circle and dark lines: Cas9/gRNA complex. Scissors in (**a**) denote insertion of cargo cassettes into DSBs on homologous chromosomes including SA, T2A, gRNA, and selection marker *mCerulean*. **b**–**d** Distinct outcomes of three DSB repair mechanisms followed by Cas9/gRNA cleavage of the homologous “receiver” chromosome. Slashes on the second exons in (**b**)–(**d**) indicate loss of function on the marked chromosomes. Labels indicate resulting phenotypes. **e, f** Mosaic clones of somatic gene conversion (SGC) in two CopyCatcher lines. Photographs show the phenotypes and fluorescence patterns of two CopyCatcher elements inserted into the intron of *white* and *ple* loci in flies without ((**e**) *w*^[3XP3-CC]^, (**f**) *ple*^[CC]^) or with ((**e**) *w*^[ATG-,3XP3-CC]^, (**f**) *ple*^[ATG-,CC]^) associated ATG^–^ point mutations, or F1 mosaics resulting from Cas9-mediated copying ((**e**) *w*^[ATG-,3XP3-CC]^/*w*+; Cas9/+, (**f**) *ple*^[ATG-,CC]^/Cas9). SGC clones generated by *ple*^[ATG-,CC]^ are outlined with white dotted lines. Rightmost panels in (**e**) and (**f**) are higher magnification views of areas delineated by white boxes in the lower magnification views immediately to their left. At least five independent flies were imaged and observed in (**e**) and (**f**) with similar results. Scale bars stand for 150 pixels in (**e**) and (**f**).

We sought to resolve the nature of somatic mutations generated through the action of gene drives using active genetic elements referred to as CopyCatchers designed to detect and quantify potential somatic copying events. CopyCatchers include a guide RNA (gRNA) for copying themselves at their site of genomic insertion into the introns of target genes and also harbor a genetic cassette that marks individual and descendent clones of cells in which these elements have been copied to the homologous chromosome. Such clones are delineated both by the expression of a fluorescent marker (DsRed) and by the creation of visible adult phenotypes (Fig. 1a). CopyCatchers also carry a *T2A-DsRed* transgene preceded by a strong splice acceptor (SA) that hijacks the original splicing of the target gene, thus generating an in-frame fusion product between endogenous gene coding sequences and the *DsRed* reporter, which is thereby expressed under the control of native *cis*-regulatory sequences. The rationale for the *DsRed* reporter gene being preceded by a T2A self-cleavage peptide is to avoid potential signal quenching that might arise in direct protein fusions with endogenously encoded peptides where protein folding of the juxtaposed domains would be unpredictable. In addition, CopyCatchers include a separate conventional dominant fluorescent eye marker (*3XP3-mCerulean*) for tracking the element in genetic crosses (Fig. 1a). In-frame fusion of the T2A-DsRed reporter with the endogenous gene also results in truncation of endogenous gene transcripts, thus generating recessive loss-of-function alleles in the target gene.

Transgenic flies carrying CopyCatchers were associated *in cis* with translation disruptive mutations upstream of the CopyCatcher insertion site to render DsRed expression conditional upon copying of the element to a wild-type chromosome. These translation abrogating ATG^–^ mutations were placed sufficiently far from the CopyCatcher insertion site so as to be outside of HDR-mediated copying range ≥1 kb from the gRNA cut site, which is well beyond the 150–200 bp range typically associated with localized directional gene-conversion events, which are initiated by 5′–>3′ resection accompanying HDR repair, followed by synthesis-dependent strand annealing (SDSA) and potential D-loop migration and resolution (Fig. 1a)^37–46^. DsRed fluorescence can be restored, however, if the elements copy themselves onto the wild-type homolog allele in a Cas9-dependent fashion thereby separating themselves from the linked ATG^–^ mutations. Such copying events would also generate homozygous mutant clones of descendent cells. Uncut alleles or  short NHEJ indels generated in the process, however, should be phenotypically silent since CopyCatchers are inserted into non-essential intronic sites (Fig. 1c, d).

We inserted CopyCatchers into three different loci: *white* (*w*), *Tyrosine 3-monooxygenase* or *pale* (*ple*), and *yellow* (*y*). As predicted, each of these CopyCatchers created recessive mutant alleles that displayed readily identifiable pigmentation defects when homozygous (Fig. 1e, f and Supplementary Fig. 1). For ease of reference, we denote these three CopyCatcher transformant lines as *w*^[CC]^, *ple*^[CC]^, and *y*^[CC]^ respectively. The three CopyCatchers also expressed the DsRed protein in patterns conforming to the endogenous targeted gene (Fig. 1e, f: lower panels, first column, and Supplementary Fig. 1a). In addition, the homozygous *w*^[CC]^ and *y*^[CC]^ CopyCatchers exhibited strong loss-of-function pigmentation phenotypes (white eyes and yellow bodies respectively, Fig. 1e: top panel first column and Supplementary Fig. 1a). Since the *ple* gene is essential for viability, the *ple*^[CC]^ CopyCatcher insertion was maintained as a balanced heterozygous stock (*ple*^[CC]^/TM6). Homozygous patches of *ple*^[CC]^/*ple*^[CC]^ mutant tissue could be generated in the presence of Cas9, and these clones displayed fully penetrant loss-of-pigmentation phenotypes as described further below (Fig. 1f: top panels, third and fourth columns).

Following the scheme outlined above, we next combined the DsRed^+^ CopyCatcher elements with 5′ translation disruptive ATG^–^ mutations and denoted these recombinant DsRed^–^ CopyCatcher alleles as *w*^[ATG-,CC]^, *ple*^[ATG-,CC]^ and *y*^[ATG-,CC]^ (Fig. 1e, f: second columns and Supplementary Fig. 1b). Heterozygous ATG^–^ CopyCatchers were tested by placing them *in trans* to a wild-type (+) allele and combining them with different Cas9 sources expressed under the control of distinct promoters inserted at different chromosomal locations. Three Cas9-dependent outcomes are possible in somatic cells of such individuals: copying to the homolog chromosome (Fig. 1b), no cutting (Fig. 1c), or generation of NHEJ-induced indels (Fig. 1d). Among these three alternatives, only “copying”, mediated by cutting at the targeted site on the receiver chromosome followed by gene conversion with CopyCatcher sequences, would separate the elements from their linked 5′ ATG^–^ mutations permitting expression of the DsRed fusion protein in these cells and their mitotic descendants (Fig. 1b). Also, as mentioned above, clonal adult tissues derived from these DsRed^+^ cells should display homozygous loss-of-function phenotypes (e.g., *w*^[ATG-,CC]^ = white eyes; *ple*^[ATG-,CC]^ = pale unpigmented bristles and thoracic epidermis; *y*^[ATG-,CC]^ = yellow cuticle) (Fig. 1e, f: third and fourth columns, Supplementary Fig. 1c). Note again, that both small NHEJ-induced indels or non-cutting events should result in DsRed^–^ and wild-type pigmentation phenotypes (Fig. 1c, d).

### Concordance of CopyCatcher SGC induced fluorescent and mutant phenotypes

Initial tests of CopyCatchers revealed that Cas9-induced SGC events were readily observed and resulted in DsRed^+^ expressing cells coinciding with adult clones exhibiting mutant pigmentation phenotypes (Fig. 1e, f and Supplementary Fig. 1). We characterized the concordance of these two CopyCatcher phenotypes further. In the case of the homozygous *y*^[CC]^ element, DsRed expression closely followed that of the endogenous *y* gene in larval epidermal cells giving rise to ventral denticle hairs (Supplementary Fig. 1a)^47^. During subsequent developmental stages (e.g., adults), however, specific fluorescence was difficult to detect since the endogenous *y* gene is only weakly expressed at these stages. As expected, combining the *y*^[CC]^ element with the upstream *y*^[1]^ allele (*y*^[ATG-,CC]^/*y*^[ATG-,CC]^) resulted in loss of the DsRed signal (Supplementary Fig. 1b). When the *y*^[ATG-,CC]^ chromosome was placed *in trans* to a wild-type X-chromosome in the presence of Cas9 (*y*^[ATG-,CC]^/+; Cas9/+), however, we observed individual DsRed^+^ cells and *y*^–^ denticles in corresponding positions (Supplementary Fig. 1c), providing proof-of-principle for the CopyCatcher concept.

We also analyzed the *w*^[CC]^ and *ple*^[CC]^ elements in greater detail with a particular interest in establishing robustly expressed DsRed reporters during adult stages that would enable facile and accurate quantification of SGC events. *ple*^[CC]^/+ (or *ple*^[CC]^/TM6) flies exhibited strong DsRed expression in epidermal nuclei throughout the adult thorax (Fig. 1f: first column, lower panel). The fluorescent signal in eyes of homozygous *w*^[CC]^ flies driven by the endogenous *w* promoter, however, was rather faint due to low levels of *white* gene expression during late phases of eye development. We, therefore, employed CRISPR editing to boost expression by inserting the artificial *3XP3* eye-specific promoter upstream of the translation initiator ATG codon of the *w* locus. When this *w*^[3XP3]^ allele was combined with the *w*^[CC]^ CopyCatcher (*w*^[3XP3-CC]^), a strong reproducible eye-specific expression of the DsRed reporter was indeed observed (Fig. 1e: first column, lower panel).

As expected, combining both the *w*^[3XP3-CC]^ and *ple*^[CC]^ elements with 5′ translation disrupting mutations eliminated their respective DsRed signals (Fig. 1e, f: second columns). When these dual mutant alleles were placed *in trans* to wild-type chromosomes in the presence of Cas9, however, DsRed reporter expression was restored in precise one-to-one correspondence with loss-of-function mutant phenotypes in individual bristles indicative of accurate SGC (Fig. 1f: third and fourth columns). In the case of *w*^[ATG-,3XP3-CC]^/+; Cas9/+ females (only females could be scored since *w* is on the X-chromosome), nearly all flies exhibited mosaic eyes with at least one eye having large white patches in a background of wild-type (red) pigmented cells. All such clonal sectors of white ommatidia also expressed DsRed (Fig. 1e: third column). Similarly, both male and female *ple*^[ATG-,CC]^/Cas9 individuals displayed numerous thoracic patches of pale bristles faithfully coinciding with underlying DsRed^+^ epidermal nuclei, indicative of precise CopyCatcher-driven SGC events and concomitant homozygous *ple* loss-of-function in these clones (Fig. 1f: third and fourth columns). The reliable concordance of fluorescence and mutant phenotypes for both the *w*^[ATG-,3XP3-CC]^ and *ple*^[ATG-,CC]^ elements validates CopyCatchers as efficient and precise SGC tracking systems.

### Quantifying SGC events with the *white* and *ple* CopyCatcher elements

**Fig. 2:**
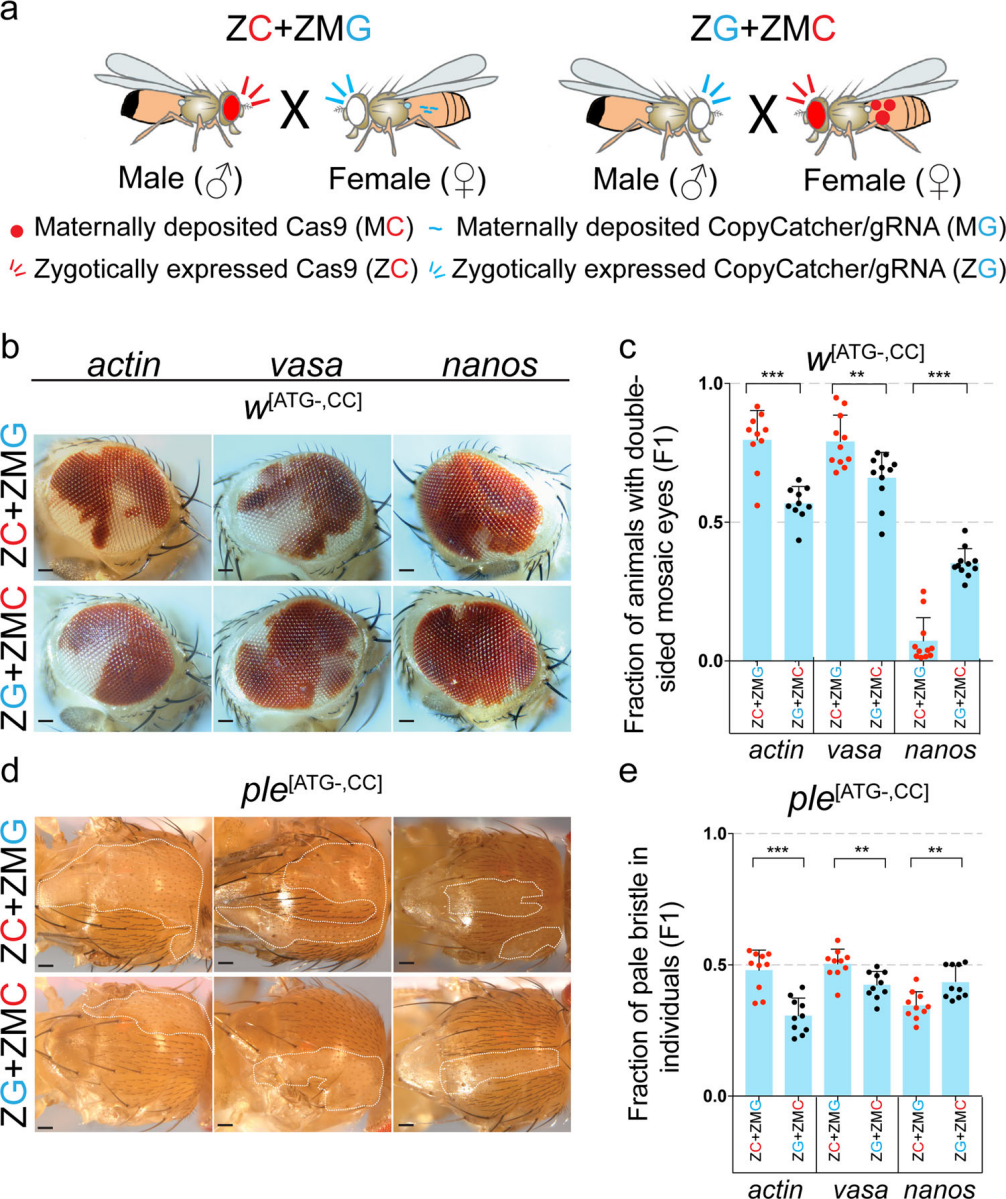
Highly efficient gene conversion in somatic cells revealed by using the CopyCatcher system. **a** Paternal and maternal crossing schemes using either F_0_ males or females carrying Cas9 transgenes inserted in the *yellow* locus expressed by different promoters and marked with *3XP3*-*DsRed* (in the eye). Trans-heterozygous F_1_ females obtained in both crosses carrying both CopyCatcher elements inserted into the *white* (*w*) or *pale* (*ple*) loci and static Cas9 expression cassettes were used to score SGC. **b**, **d** Examples of mosaic phenotypes (*n* = 15 biological independent flies were observed) of the F_1_ trans-heterozygous *y*^+^, *w*^[ATG-,CC]^/*y*^[Cas9]^, *w*^+^ compound eyes (**b**) or *y*^[Cas9]^/+; *ple*^[ATG-,CC]^/+ thorax bristles (**d**). Clones of pale bristles delineated by dotted white lines in (**d**) denote the patches created by SGC events. **c**, **e** SGC rates measured by the fraction of F_1_ female progeny having double-sided mosaic eyes (**c**), or the fraction of pale thorax bristles relative to the total thorax bristles in individual F_1_ trans-heterozygous females (**e**). Each dot in (**c**) represents SGC averages from single vials (*n* = 10 biological independent crosses for *actin* promoter-driven Cas9, and *n* = 11 biological independent crosses for both *vasa* and *nanos* promoter-driven Cas9) and in (**e**) represents the percent of pale bristles for an individual fly (as shown in panel (**d**), *n* = 10 biological independent flies). ZC+ZMG: Cas9 males crossed with CopyCatcher females, ZG+ZMC: CopyCatcher males crossed with Cas9 females. Error bars indicate mean values ± SD. Asterisks represent significance with a two-tailed *t*-test: three asterisks (*p* < *0.001*), two asterisks (*p* < *0.01*), one asterisk (*p* < *0.05*), and ns (not significant). Scale bars stand for 200 pixels. Raw data for (**c**) and (**e**) are provided as Source Data files.

*Drosophila* offers flexible genetic tools for dissecting and optimizing genetic processes such as assessing how tissue specificity, timing, or levels of Cas9 expression might affect rates of CopyCatcher induced SGC events. We placed *w*^[ATG-,CC]^ and *ple*^[ATG-,CC]^ CopyCatcher double *cis*-mutant allelic combinations *in trans* to wild-type alleles and evaluated SGC efficiency by semi-quantitative (*w*^[ATG-,CC]^) and quantitative (*ple*^[ATG-,CC]^) measures when using three different X-linked sources of Cas9: *actin*-Cas9 (ubiquitously expressed during the whole developmental stages)^48^, *vasa*-Cas9 (expressed primarily in germline cells at all developmental stages, as well as embryonic somatic gonadal precursor cells)^49^ and *nanos*-Cas9 (specifically transcribed in the nurse cells with the mRNA localized to the posterior pole of oocytes and embryos)^50^. We conducted crosses in which the Cas9 transgene was provided either paternally (denoted Zygotic Cas9 or ZC, Fig. 2a and Supplementary Fig. 2a, b) or maternally (denoted as Zygotic plus Maternal Cas9 or ZMC, Fig. 2a and Supplementary Fig. 2a, b), and determined how differing temporal and spatial patterns, as well as levels of Cas9, might impact rates of SGC (Supplementary Fig. 2a, b). In the case of the *w*^[ATG-,CC]^ element, we employed the original *w*^[ATG-,CC]^ line (without the inserted 5′ *3XP3* artificial promoter) to perform the clonal analysis since the low levels of DsRed driven by the endogenous *w* promoter do not interfere with scoring the Cas9-associated DsRed selection marker common to the three Cas9 lines tested (all of these Cas9 elements were inserted into the same target site in the *y* locus).

Virtually 100% of F_1_ CopyCatcher females carrying Cas9 (*y*^+^*w*^[ATG-,CC]^/*y*^[Cas9]^*w*^+^ and *y*^[Cas9]^/+; *ple*^[ATG-,CC]^/+ genotypes) displayed extensive mosaic mutant phenotypes, for all sources of Cas9 and for both paternal and maternal crossing schemes (Fig. 2b, d). These results confirm that SGC is surprisingly frequent, occurring with complete penetrance and that the *w*^[CC]^ and *ple*^[CC]^ CopyCatchers are highly efficient in detecting such copying events. We analyzed the extent of SGC events by scoring the fraction of flies having mosaic patches in both eyes of *y*^+^*w*^[ATG-,CC]^/*y*^[Cas9]^*w*^+^ females (a semiquantitative index) (Fig. 2c), and by tabulating the fraction of enumerated pale thoracic bristles for *y*^[Cas9]^/+; *ple*^[ATG-,CC]^/+ individuals (a quantitative index) (Fig. 2e).

In paternal Cas9 crosses (ZC♂ + ZMG♀, Fig. 2a), both *y*^+^*w*^[ATG-,CC]^/*y*^[Cas9]^*w*^+^ and *y*^[Cas9]^/+; *ple*^[ATG-,CC]^/+ females exhibited SGC frequencies that increased according to inferred somatic Cas9 levels, with mosaic patches occurring most frequently in the following order of promoter-driven Cas9 expression: *actin* > *vasa* > *nanos* (Fig. 2b–e). This trend was also mirrored in F_1_ progeny from maternal crossing schemes (ZG♂ + ZMC♀, Fig. 2b–e). For paternal crosses in which Cas9 was driven by the *actin* and *vasa* promoters, ~80% of *y*^+^*w*^[ATG-,CC]^/*y*^[Cas9]^*w*^+^ individuals displayed *w*^*–*^ clones in both eyes. Notably, however, maternal crossing schemes for supplying Cas9 resulted in significantly lower SGC frequencies in F_1_ CopyCatcher progeny (e.g., 57% for *actin*-Cas9 and 66% for *vasa*-Cas9, between ZC+ZMG and ZG+ZMC crosses with *vasa*-Cac9, *p* = 0.0035, two-tailed *t*-test) than observed for paternal crossing schemes employing the same Cas9 sources (Fig. 2b, c). The reductions in SGC associated with maternal versus paternal pedigrees were particularly evident when crossing CopyCatcher males to *actin*-Cas9 females (the countervailing exception of *w*^[ATG-,CC]^ or *ple*^[ATG-,CC]^ males crossed to *nanos*-Cas9 females was analyzed in the Supplementary Information, Supplementary Fig. 3). A similar trend was evident for *y*^[Cas9]^/+; *ple*^[ATG-,CC]^/+ females, in which about half of all thoracic bristles displayed pale phenotypes for the *actin*-Cas9 and *vasa*-Cas9 source (SGC percentages were respectively: 48% and 50%) in paternal crosses versus 31% and 42%, respectively for the corresponding maternal crosses (Fig. 2e, *vasa*-Cac9: *p* = 0.0033, *nanos*-Cac9: *p* = 0.0036, two-tailed *t*-test). One explanation for these results is that SGC frequencies increase with overall Cas9 levels but decrease in response to maternal accumulation of Cas9/gRNA complexes in the egg, which can act at an early developmental stage to induce cleavage resistant NHEJ alleles precluding SGC during subsequent stages. Several other crossing schemes further support the hypothesis that higher levels of Cas9 delivered at later developmental stages optimize SGC (Supplementary Figs. 3 and 4).

The differing rates of SGC observed in paternal versus maternal CopyCatcher crossing schemes raised the possibility that the ability of the gRNAs to gain access to their chromosomal targets might differ between these two crossing schemes. We addressed this possibility in two ways. First, we tested CopyCatcher elements for efficiency of germline transmission, which provides a standardized measure for HDR-mediated DSB repair in meiotic lineages. In these experiments, F_1_ trans-heterozygous *y*^+^*w*^[ATG-,CC]^/*y*^[Cas9]^*w*^+^ or *y*^[Cas9]^/+; *ple*^[ATG-,CC]^/+ females were crossed to *w*^*118*^ males (Supplementary Fig. 2a, b, and Supplementary Fig. 4a, b). Among F_2_ progeny from both paternal and maternal crossing of *w*^[ATG-,CC]^, over 90% of individuals were positive for both CFP (the dominant CopyCatcher marker) and the white eye phenotype, representing a composite of both donor and receiver chromosomes carrying the CopyCatcher elements (Supplementary Fig. 4a). Similarly, among F_2_ progeny of *ple*^[ATG-,CC]^ crosses, at least 82% flies were CFP^+^ and 42% flies (= 84% germline gene conversion) were RFP^+^ throughout the thorax (denoted by RT in composite Supplementary Fig. 4b), which selectively scored transmission of the HDR converted receiver chromosome (Supplementary Fig. 4b). In contrast, the static RFP-marked Cas9 element, serving as an internal control, displayed standard Mendelian transmission (~50% inheritance). These results indicate that both gRNAs employed in the *w*^[CC]^ and *ple*^[CC]^ CopyCatchers sustain efficient target cleavage and HDR-mediated copying in the germline.

As a complementary approach, we performed next-generation sequencing (NGS) on genomic DNA samples from two typical CopyCatcher crossing schemes using the *vasa*-Cas9 source (Supplementary Fig. 5). In this analysis, the fraction of uncut wild-type alleles was <5% of the total alleles recovered on the non-converted target chromosomes (the remainder were NHEJ indels, which differed in prevalence and abundance of specific alleles based on the crossing scheme). These NHEJ events altered sequences at varying distances from the gRNA cutting sites, but even the largest lesions did not extend into neighboring exons (Supplementary Figs. 5a, b, e, f). Collectively, these findings suggest that the gRNAs carried by the *w*^[CC]^ and *ple*^[CC]^ CopyCatchers are highly efficient in cutting target chromosomes and that the differing rates of SGC observed in various crossing scenarios can most likely be attributed to particular developmental patterns and levels of Cas9 expression. We hypothesize that these variations in Cas9 expression determine a balance between NHEJ (dominating during early embryonic stages of development) and somatic HDR-mediated repair (acting later during germline development, see Supplementary Information for further supportive evidence for this hypothesis based on in-depth analysis of SGC efficiencies observed in a variety of different crossing schemes).

### A targeted screen identifies genes influencing CopyCatcher-induced SGC events

Efforts to boost levels or activity of key HDR pathway components or to reduce the activities of competing NHEJ components typically produce modest increments in HDR/NHEJ ratios in mammalian cells, but still fall short of the efficiencies required for many potential applications. It is unclear, however, whether other components involved in DNA repair or chromosome pairing also contribute to such inter-chromosomal somatic correction. We speculated that factors altering rates of SGC might similarly impact HDR efficiencies in mammalian contexts.

**Fig. 3:**
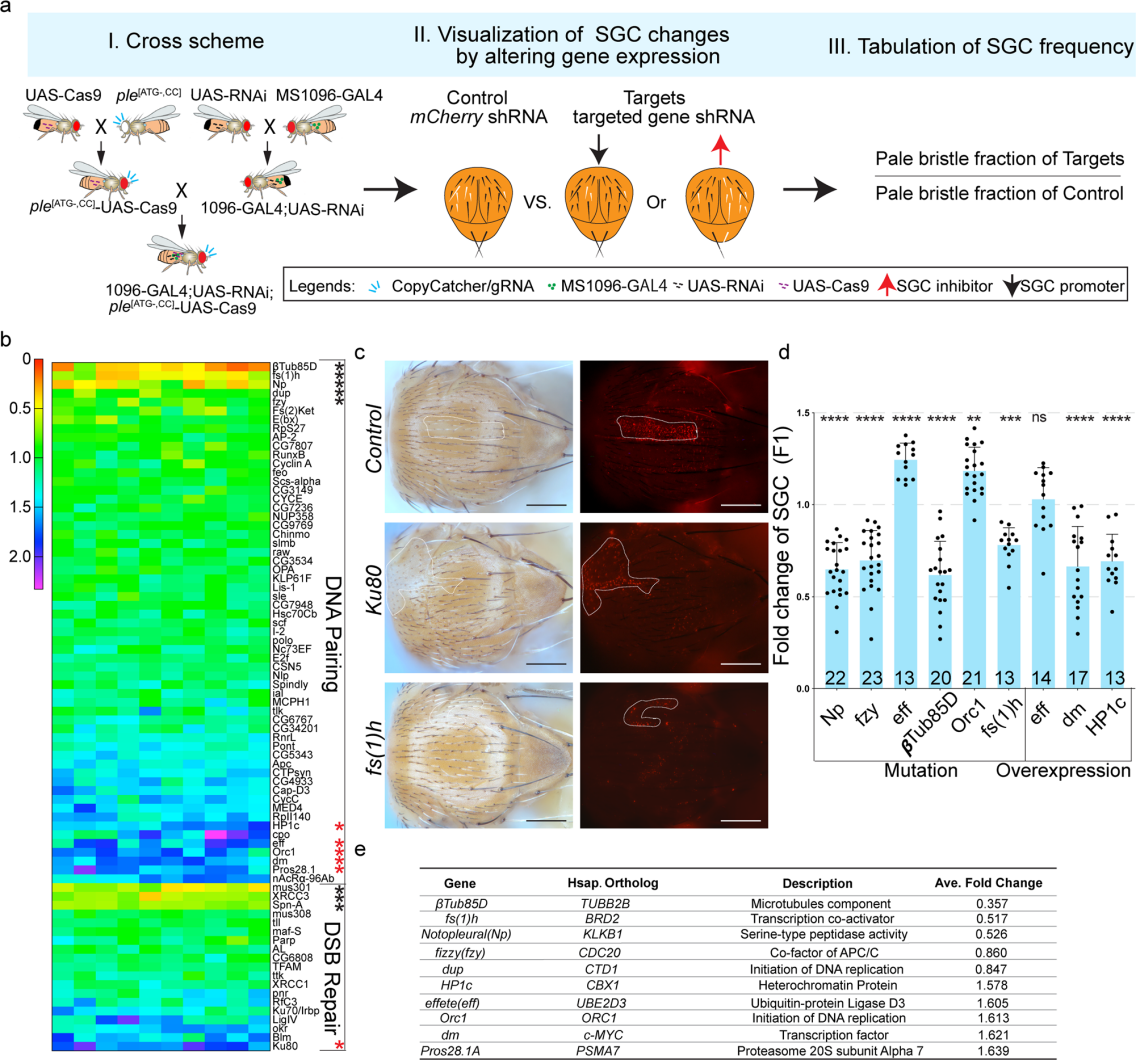
Genetic screen for SGC modifiers using CopyCatcher. **a** Workflow of genetic RNAi screen using *ple*^[ATG-,CC]^. **b** Heatmap displays results for genes modulating *ple*^[ATG-,CC]^ induced SGC. Values are shown as fold change of SGC frequency by normalizing the pale bristles on every single fly to averaged control flies. The value is calculated by dividing the SGC frequency with knock-down indicated genes by the average control SGC frequency obtained with an shRNA targeting *mCherry*. Scores less than 1 indicate genes promoting SGC (black stars indicate the top SGC promoters) while scores greater than 1 represent inhibitors of SGC (red stars indicate the top SGC inhibitors). **c** Examples of knocking down SGC inhibitor (*Ku80*) and promoter (*fs(1)h*) (Ten independent flies were imaged as showed in Supplementary Fig. 6). The dominant SGC patches with light thoracic pigmentation and pale bristles are delineated with dotted white lines. Scale bars indicate 100 pixels. **d** Validation of the top SGC modulating candidates in individuals carrying either heterozygous loss of function alleles or MS1096-GAL4 and UAS over-expression constructs. Each dot represents the relative fraction of pale bristles scored for a single fly and the number of flies counted was labeled at the bottom of each bar. *P* values were: *Orc1* = 0.0011 (**, one-way ANOVA), *fs(1)h* = 0.0006 (***, one-way ANOVA), *eff* = 0.9969 (ns, one-way ANOVA), and others are <0.0001 (****, one-way ANOVA). Error bars indicate mean ± S.D. **e** Key gene information for the top SGC modifiers identified by the *ple*^[ATG-,CC]^ CopyCatcher RNAi screen (in panel (**b**)). Raw data for (**d**) is provided as a Source Data file.

As a first step in defining factors that influence SGC, we screened 109 of the *Drosophila* TRiP RNAi collection, to determine whether any of these genes when knocked down, might lead to differing rates of *ple*^[ATG-,CC]^-mediated SGC events (Fig. 3a)^51,52^. Targeted expression of the various RNAi lines was induced using the GAL4/UAS transactivation system (Fig. 3a)^53^. We chose the *ple*^[ATG-,CC]^ CopyCatcher for these experiments since it was best suited for the quantification of SGC events. We surveyed a set of 77 DNA pairing factors (DNA pairing) and 32 genes associated with DSB repair pathway factors (DSB repair, Fig. 3b). As positive controls, we included *Irbp* (*Drosophila Ku70* ortholog), *Ku80*, and *DNA-ligIV*, which we predicted should increase SGC and *Spn-A* (*Drosophila Rad51* ortholog), which ought to decrease SGC in response to RNAi knock-down.

We screened candidate SGC modifiers by generating test females carrying the *ple*^[ATG-,CC]^ CopyCatcher element, the strong thorax and wing-specific MS1096-GAL4 driver, and UAS-RNAi cassettes (whose expression is induced by the GAL4 trans-activator) and scored the fraction of pale bristles in individuals expressing the UAS-RNAi construct relative to controls (Fig. 3a). As a control, we assessed the efficiency of an shRNA targeting the *mCherry* coding sequence. For each RNAi line tested (at least three independent crosses per RNAi line), we averaged the fraction of pale thoracic bristles in 15 control flies and in ≥10 flies of each RNAi genotype. We tabulated relative SGC frequencies (displayed as a heat map) by calculating the fold change of SGC events for each RNAi line relative to the batch *mCherry* RNAi controls (Fig. 3a, b). Among our four predicted positive RNAi lines, knock-down of *Ku80* exhibited the strongest SGC-stimulating effects, with SGC increasing 1.75-fold over controls, while *Irbp* and *DNA-ligIV* knock-down demonstrated significant but more modest, increases in SGC frequency (Fig. 3b, c). In contrast, down-regulation of *Spn-A* (*Rad51*) decreased the rate of SGC to 0.68-fold of control levels (Fig. 3b). These predicted effects of inhibiting known DSB repair components validated the CopyCatcher system as an effective in vivo genetic screening platform. Next, we screened the remaining 109 candidate genes implicated in DNA pairing and DSB repair, and identified *Pros28.1* (1.64-fold), *dm* (1.62-fold), *Orc1* (1.61-fold), *eff* (1.61-fold), and *HP1c* (1.58-fold) as loci inhibiting SGC (i.e., SGC was increased by RNAi of these genes) and *βTub85D* (0.36-fold), *fs(1)h* (0.52-fold), *Np* (0.53-fold), *fzy* (0.86-fold) and *dup* (0.85-fold) as promoters of SGC in *Drosophila* (i.e., SGC was decreased by RNAi of these genes, Fig. 3b, c, e, Supplementary Fig. 6)^54–65^.

Since RNAi typically results in a reduction, but not elimination, of gene activity, we wondered whether simply reducing the gene dose of SGC candidate modifiers by 50% in heterozygous mutants might mimic the effect of RNAi. We confirmed that heterozygous loss-of-function alleles of the *Np* (0.65-fold), *fzy* (0.7-fold), *βTub85D* (0.62-fold), and *fs(1)h* (0.78-fold) loci decreased SGC frequency while lowering the dosage of the *eff* (1.24-fold) and *Orc1* (1.18-fold) genes enhanced SGC significantly (Fig. 3d). Another way to assess the roles of candidate genes in promoting or antagonizing SGC is to overexpress them, which according to simple models would be predicted to have the opposite effect to knocking them down by RNAi (Fig. 3d). We found this was indeed the case for *HP1c* (0.69-fold) and *dm* (0.67-fold) genes for which UAS-overexpression transgenes were available. Collectively, this analysis supports the use of CopyCatchers as useful tools to identify and evaluate SGC modifiers in vivo.

### Functional conservation of SGC candidate genes for DSB repair in human cells

As indicated above, a significant bottleneck in applying CRISPR/Cas9 technologies to gene and cell therapies is the pronounced preference of somatic cells for repairing DSBs via NHEJ rather than HDR. We wondered whether human orthologs of genes modulating SGC in *Drosophila* would also influence rates of somatic HDR in human cells. For this purpose, we generated a fluorescent-based reporter system capable of simultaneously quantifying NHEJ and HDR events in HEK293T cells (Supplementary Fig. 7a, b). This system consists of a stable human epithelial kidney cell-line expressing a single copy of a *P2A-copGFP* cassette inserted into the 3′ terminal region of the *GAPDH* gene (the second allele on the homolog chromosome carries an NHEJ-induced point mutation, which is targeted by gRNA^NHEJ^). We could thus measure both plasmid and homolog chromosome-templated DSB repair using this heterozygous HEK293T *GAPDH-copGFP* cell line (Supplementary Fig. 7a, b).

**Fig. 4:**
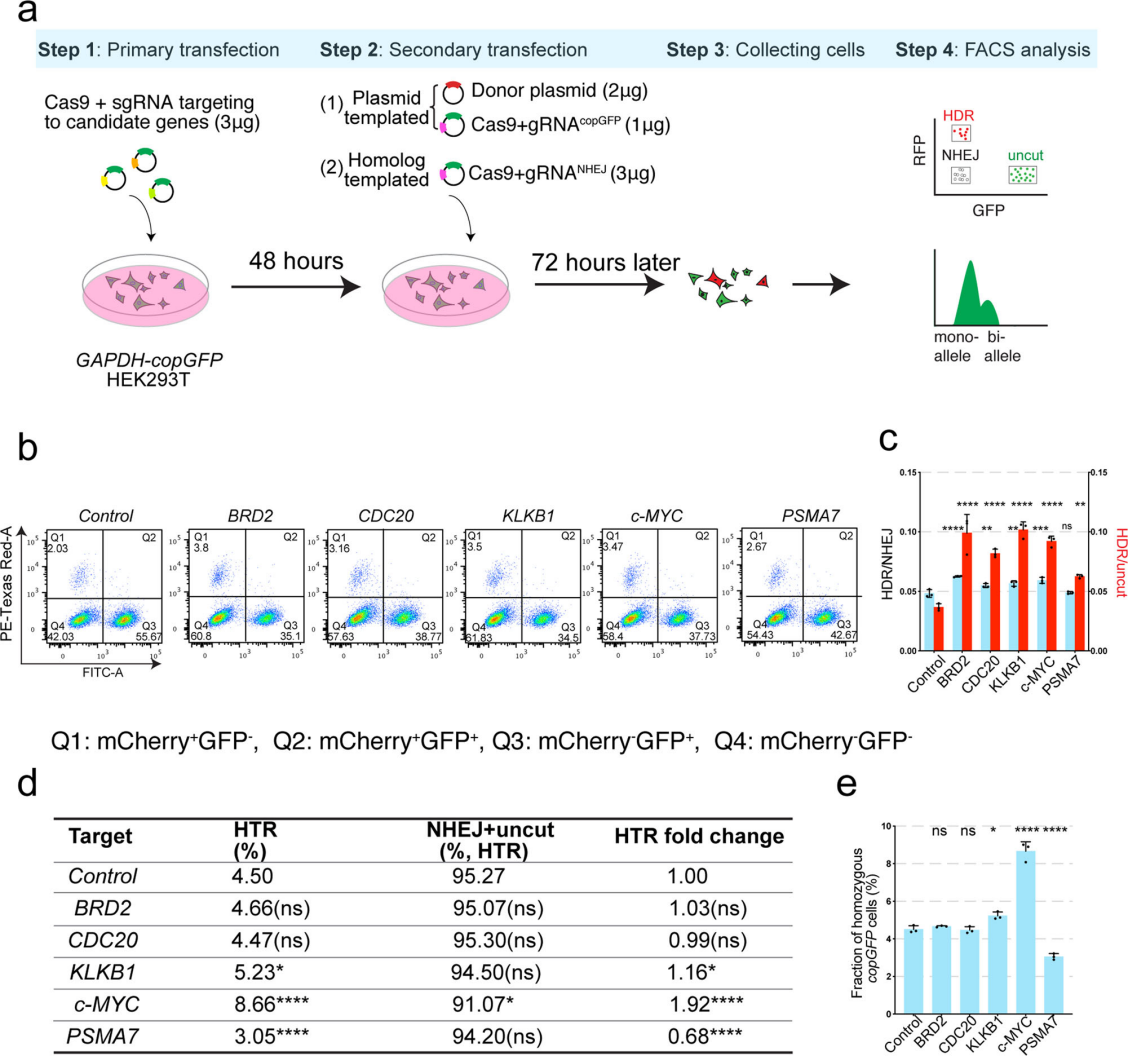
Application of *Drosophila* SGC modifiers in mammalian cell lines. **a** Workflow for testing HDR rates resulting from RNAi knock-down of human orthologs of top hits modifying *Drosophila* SGC in human *GAPHD-copGFP* cells. Both exogenous plasmid and homologous chromosome templated somatic HDR were quantified with a GFP fluorescent readout. Three biological replicates were conducted. **b** FACS plots with donor plasmid-mediated DSB repair. The four quadrants correspond to the following editing outcomes: Q_1_: mCherry^+^ GFP^-^ = HDR, Q_2_: mCherry^+^ GFP^+^ = none, Q_3_: mCherry^-^ GFP^+^ = uncut, Q_4_: mCherry^-^ GFP^-^ = NHEJ. The average frequency of each event was labeled. **c** Histogram of plasmid-mediated HDR/NHEJ ratio (light blue bars) and HDR/uncut ratio (red bars) in *GAPDH-copGFP* heterozygous cell line with or without knock-down candidate SGC modifier homologs. Three independent experiments were performed. *p* Values for HDR/NHEJ ratio: *BRD2* < 0.0001 (****, one-way ANOVA), *CDC20* = 0.0086 (**, one-way ANOVA), *KLKB1* = 0.0016 (**, one-way ANOVA), *c-Myc* = 0.0002 (***, one-way ANOVA), *PSMA7* = 0.9935 (ns, one-way ANOVA), *p* values for HDR/uncut ratio: *BRD2* < 0.0001 (****, one-way ANOVA), *CDC20* < 0.0001 (****, one-way ANOVA), *KLKB1* < 0.0001 (****, one-way ANOVA), *c-Myc* < 0.0001 (****, one-way ANOVA), *PSMA7* = 0.0044 (**, one-way ANOVA). **d** Table indicating HDR frequency employing the homologous chromosome as repair template following knock down of potential SGC modifiers. HTR: homolog chromosome templated recombination. *P* values for HTR were *BRD2* = 0.9165 (ns, one-way ANOVA), *CDC20* = 0.9997 (ns, one-way ANOVA), *KLKB1* = 0.201 (*, one-way ANOVA), *c-MYC* < 0.0001 (****, one-way ANOVA), *PSMA7* < 0.0001 (****, one-way ANOVA), and *p* values for NHEJ + uncut were: *BRD2* = 0.9997 (ns, one-way ANOVA), *CDC20* > 0.9999 (ns, one-way ANOVA), *KLKB1* = 0.9486 (ns, one-way ANOVA), *c-Myc* = 0.0178 (*, one-way ANOVA), *PSMA7* = 0.8412 (ns, one-way ANOVA). **e** Histogram of fraction of homozygous *copGFP* cells undergoing HTR. Three biological independent replicates were performed. Error bars indicate mean ± S.D. Raw data for (**c**) and (**e**) are provided as Source Data files.

Plasmid-templated DSB repair was assayed by transfecting HEK293T *GAPDH-copGFP* cells with a combination of plasmids expressing *SpCas9*, a gRNA targeting to *copGFP* (gRNA^copGFP^), and a promoter-less *mCherry* donor DNA template with homology arms flanking the *copGFP* gRNA cut site (Fig. 4a and Supplementary Fig. 7a). In these traffic-light style experiments, loss of the GFP signal (Phase Q4 in FACS plots: GFP^–^ mCherry^–^) defines the fraction cells in which NHEJ events have mutated the *GFP* target gene, while concomitant gain of mCherry fluorescence (Phase Q1 in FACS plots: GFP^–^ mCherry^+^) reflects HDR events (Fig. 4b). Plasmids encoding Cas9 and gRNAs targeting candidate SGC modifiers were transfected into cells, and 2-days later we performed a second co-transfection with the *mCherry* donor plasmid and a plasmid encoding Cas9 and a gRNA targeting *copGFP*. After 72 h, cells were harvested and analyzed by FACS (Fig. 4a). We tested the human orthologs of the top 5 promoters or inhibitors of SGC identified in the fly RNAi screens. These human cell experiments confirmed that down-regulation of *BRD2* (ortholog of *Dmelfs(1)h*), *CDC20* (*Dmelfzy*), *KLKB1* (*DmelNP*), *c-MYC* (*Dmeldm*), and *PSMA7* (*Dmelpros28.1*) increased both NHEJ and HDR significantly (Figs. 3e,  4b, and Source Data). While there was a concordance of effects between the mammalian cells and *Drosophila* regarding the direction of effects on HDR and SGC for the *c-MYC* and *PSMA7*, we observed alterations of opposite sign to those identified in *Drosophila* for *CDC20*, *BRD2* and *KLKB1* (Fig. 4b, c). Notably, knock-down of *c-MYC*, resulting in approximately a 50% reduction in mRNA levels, increased the proportion of HDR-mediated fluorescence marker swapping relative to uncut *GAPDH* targets by 2.5-fold, and increased the average ratio of HDR/NHEJ on average by 23% (Fig. 4b, c, Source Data, Supplementary Fig 7d). This enhanced cassette copying might potentially be augmented further by a more complete disruption of *c-MYC* expression. Thus, these pilot experiments identified inhibition of *c-MYC* as a prime candidate for augmenting exogenous DNA-templated somatic HDR events in this system.

We conducted further HDR analysis in HEK293T *GAPDH-copGFP* cells employing the homologous chromosome as the DSB repair template. In this system, no exogenous DNA template was included. Instead, a gRNA targeting the NHEJ allele present on the homologous *GAPDH* allele was provided, thus creating a genome repair context similar to that of CopyCatchers in *Drosophila* (Supplementary Fig. 7b). We scored the fraction of cells that were homozygous versus heterozygous for the *GADPH-copGFP* allele by quantitative fluorescence-activated cell sorting (FACS), which can distinguish cells carrying one versus two copies of *GFP* (see multi-level validation analysis presented in Supplementary Fig. 8b–d and summarized below). Using this mono- versus bi-allelic *copGFP* assay, we then knocked down levels of the *BRD2*, *CDC20*, *KLKB1*, *c-MYC*, and *PSMA7* genes, all of which influenced rates of plasmid-templated HDR (Fig. 4b). Once again, *c-MYC* behaved as an SGC inhibitor as revealed by an increased rate of homolog-templated HDR (1.92-fold relative to controls, Fig. 4d, e, Source Data, Supplementary Fig. 8a). In addition, knocking-down *KLKB1* and *PSMA7* significantly altered the fraction of homozygous *GFP*, but these effects were opposite to those observed in *Drosophila* (Fig. 4d, e, Source Data, compare to Fig. 3b, c).

The inferred genotypes of FACS sorted homozygous *copGFP* cells were verified by single-cell cloning. We isolated single cell colonies from control (19 colonies) and *c-MYC* knock-down (17 colonies) treatments and verified the homozygosity of these isolated single colonies by amplifying their genomic DNA with primers flanking the insertion site which could distinguish the longer insertion allele fragment from that of the shorter NHEJ allele (Supplementary Fig. 8b). Only the longer *copGFP* bearing fragment was amplified from the bi-allelic *GAPDH-copGFP* cells, while amplification from a few mis-gated heterozygous *GAPDH-copGFP* cells revealed both long and short amplicon fragments (Supplementary Fig. 8b). In validation of our stringent fluorescent gating protocol, 76% of the *c-MYC* knock-down clones (13 of 17 colonies) were verified as bi-allelic for the *copGFP* insertion, as were 74% (14 of 19 colonies) of the control colonies (Supplementary Fig. 8b). Consistent with the inference from PCR analysis that clones amplifying only the single large band were homozygous for the *copGFP* element, the relative transcription level of *copGFP* was approximately double in these putative homozygous colonies compared to clones scored as being trans-heterozygous for the original *copGFP* and NHEJ allele (Supplementary Fig. 8c).

As further validation of cells interpreted as having undergone homolog-based repair with both chromosomes carrying the *copGFP* insertion of interest, we identified a closely linked polymorphism associated with the gRNA cleavage site (an SNP 94 bp from the cut site) (Supplementary Fig. 8d). We used primers from the *copGFP* element and adjacent genomic sequence to amplify and Sanger sequence a short fragment containing this SNP and the *copGFP* gene from inferred homozygous and heterozygous colonies (Supplementary Fig. 8d). In three putative homozygous colonies (which also displayed elevated *copGFP* expression, colonies 2, 6, and 25), we observed equivalent double peaks at the SNP site, confirming that indeed both alleles were associated with the *copGFP* insertion. Similar analysis of three control heterozygous colonies (1, 9, and 23) revealed only a single peak, indicating that no HDR occurred in these colonies. In one candidate homozygous clone (colony 34) we also observed a mono-allelic *copGFP* transgene. Given that the gRNA cut site was close to the SNP (94 bp), we imagine that in this clone localized gene conversion during the HDR repair process copied the donor polymorphism along with the *copGFP* element in this clone consistent with such islands of sequence conversion often extending 100–200 bp on either side of a DSB (Supplementary Fig. 8d)^66^. In aggregate, these data strongly support the hypothesis that a great majority of the sorted cells we scored as being homozygous do indeed carry two copies of the *copGFP* allele and that RNAi knock-down of the *c-MYC* gene increases the frequency of such events by nearly twofold.

## Discussion

In this study, we develop CopyCatchers as robust genetic systems for detecting and quantifying SGC in *Drosophila*. Strategies for manipulating somatic DSB repair mechanisms have been extensively explored in mammalian cell lines, particularly with regard to treatments increasing the rate of HDR. Two frequently used systems are the direct-repeat GFP reporter and traffic-light switches^67–70^, which rely on either phenotypic or fluorescent outputs to score somatic HDR events. However, both of these reporters are difficult to adapt for high-throughput applications based on their high false-positive rates and complex repair outcomes^68,69^. CopyCatchers offer an efficient alternative in vivo approach for detecting and quantifying SGC events.

CopyCatchers are modified active genetic elements that incorporate several unique design features to serve as single-cell resolution reporters of HDR-mediated copying events in somatic cells^2,71^. These recording elements are inserted into introns such that localized NHEJ induced indels are expected to exhibit no phenotypic effect, excluding potential false-positive signals, consistent with the observed strict concordance between fluorescent reporter and targeted loss-of-function phenotypes. DNA deep sequencing confirmed that the NHEJ events disrupt sequences at varying distances from the gRNA cut site, but do not extend into adjacent exons. CopyCatchers carry a highly sensitive fluorescent reporter gene *DsRed* fused in-frame with targeted genes whose translation was abrogated by associated 5′ translation disruptive mutations. Thus, *DsRed* expression can only be recovered if a CopyCatcher element copies itself onto a wild-type homolog chromosome thereby separating itself from the 5′ mutation. We note that the CopyCatcher insertion sites were chosen to be sufficiently distant from the 5′ mutations to preclude co-copying with the CopyCatcher allele. In addition to reanimating *DsRed* expression, copying events also generate bi-allelic mutant cells, the clonal mitotic descendants of which all displayed concordant DsRed^+^ and homozygous loss-of-function phenotypes of the target gene.

The most striking result revealed by CopyCatchers is their unexpectedly high rate of SGC events in the *white* and *ple* loci. Qualitative (*w*^[ATG-,CC]^) and quantitative (*ple*^[ATG-,CC]^) assessments of SGC frequencies were in the range of 30–50% of cells in the targeted tissues (eye and thorax respectively). These observations suggest that a substantial fraction of somatic cell phenotypes produced by gene-drive systems in which the Cas9 activity is not strictly limited to the germline are likely to be caused by homozygosity of the drive element^1,2,12^. Furthermore, by testing a variety of crossing schemes and promoters driving Cas9 expression, we identified particularly favorable configurations for promoting SGC (e.g., the paternal transmission of either broadly expressed Cas9 alone or together with the CopyCatcher), which could be enhanced yet further by altering the activities of several genes associated with somatic DSB repair (see more detailed discussion below). Thus, far from being inefficient, HDR-mediated SGC employing the homologous chromosome as a correction template can serve as a frequent repair pathway in somatic cells of *Drosophila*. CopyCatcher elements also displayed efficient germline transmission. These quantitative and single-cell resolution findings are consistent with prior reports of frequent I-*Sce*I-induced repair of a *mini*-*white* transgene using neighboring sequences located *in cis* on the same chromosome in the germline^72,73^, and with qualitative evidence for modest levels of bulk targeted cleavage noted in the soma^72–74^, although it remains unclear whether there might be mechanistic differences distinguishing cis- versus homolog-templated repair (HTR)^75^. We offer the term HTR to refer to the mechanism underlying such SGC events.

Because mammalian chromosomes do not typically engage in inter-homolog pairing as pervasively as *Drosophila*, a potential concern could be that the high efficiency of HTR-driven SGC we observed in *Drosophila* would prove less efficient in somatic cells of other organisms in which chromosome pairing is less prominent^52,76–78^. We note, however, that there is evidence in mammalian cells that the absence of chromosome pairing is due in part to an active anti-pairing process, suggesting that these cells too might be induced to engage in efficient pairing by interfering with this suppressive process^24^. Furthermore, homologous chromosome segments are actively recruited to DSBs located in transcribed regions of the genome during diploid phases of the cell cycle, indicating that HTR actively contributes to maintaining cell viability^78–80^. Indeed, in some cancer cell lines, specific chromosome arms have been found to be consistently paired along their length^24,79,80^. Also, in LOH mutants, the frequency of repairing DSB by copying from the homologous chromosome is greatly elevated^27,28^. Also, results reported in this study indicate that HTR can be detected in a human cell line and that factors influencing such SGC events in *Drosophila* (e.g., *c-MYC*) can similarly modify HTR in the human cell model.

The potential concerns discussed above regarding the potential role of chromosome pairing in mammalian cells notwithstanding, there is a great need to overcome inefficient Cas9-based genome editing in human cells since this technical impediment hampers the development of potential therapeutic tools for treating human somatic diseases^81,82^. To tackle this critical problem, various studies have pursued strategies of: inhibiting core components of the NHEJ machinery^39,83–85^; stimulating the HDR pathway^86,87^; synchronizing the cell cycle^88^; concentrating DSB repair templates at the cutting site^89^; or manipulating DSBs repair pathways in favor of HDR over NHEJ^90^.

Because CopyCatchers provide exceedingly sensitive readouts for SGC, we tested whether they might also serve as tools to probe the genetic requirements for biasing somatic cell DSB repair in favor of HDR. In a pilot genetic screen of 109 factors either being essential for DNA pairing or associating with known DSB repair factors, we identified *Orc1*, *HP1c*, *eff*, *Pros28.1*, and *dm* as loci inhibiting SGC and *βTub85D*, *fzy*, *Np*, *dup*, and *fs(1)h* as promoters of SGC in *Drosophila*. Among these candidates, we found *c-MYC*, the human ortholog of *Drosophila dm*, functions as an SGC inhibitor during both plasmid and HTR in mammalian HEK293T cells, while *BRD2*, *CDC20*, and *KLKB1* enhanced SGC, confirming that CopyCatchers can serve as preliminary screening tools for genetic modifiers of SGC. These results suggest that the identified components modulating SGC in flies (*dm*) are also relevant to both plasmid and HTR in mammalian cells, although the details of how they do so may differ between systems since in some cases we observed opposite effects of SGC between flies and mammalian cells, which may reflect distinct mechanisms or components acting in these repair processes. In future studies, it will be important to test these strategies for increasing SGC rates in additional mammalian cell lines and primary cells. Such investigations may identify additional factors or pathways that can be manipulated to bias somatic DSB repair choice, as well as small molecules influencing the choice DSB repair pathway in favor of HTR in somatic cells^80,81^. Ultimately, these HTR-based systems could provide strategies to devise precision gene therapy.

## Methods

### DNA manipulations and constructions of CopyCatcher elements

One-step assembly with NEBuilder HiFi DAssembly Master Mix was used for cloning of CopyCatcher plasmids^91^. As depicted in Fig. 1a, left and right homologous arms for each locus flanking gRNA cleavage site were amplified from the *w*^118^ wild-type fly genomic DNA. The SA-T2A sequence was amplified from *pBS-KS-attB2-SA-T2A-3XGal80-Hsp70* series plasmids (Addgene 62951 and 62952)^92^, gRNAs-expressing cassette targeting to the first intron of *yellow*, *white*, and *ple* were assembled following online published protocols (CRISPR fly *Design*-http://www.crisprflydesign.org/) and *mCerulean* selection marker was amplified from Addgene 27795 plasmid. *DsRed* was put at the downstream of T2A to indicate the copying events by fluorescence. Following assembly, CopyCatcher plasmids were transformed into NEB 5-alpha chemical competent *E. coli* cell (NEB C2987). Positive plasmids verified by sequencing were purified with Qiagen Plasmid Midi Kit (#12191), mixed with *pBS-Hsp70-Cas9* (Addgene 46294) at 500 ng/μl and 250 ng/μl each, and sent to Rainbow Transgenic Flies, Inc. for injection into *w*^118^ wild-type (*y*^[CC]^ and *ple*^[CC]^) or *Oregon-R* (*w*^[CC]^) embryos. By screening the CFP fluorescent eye-marker phenotype, male transformants carrying the *y*^[CC]^, *w*^[CC]^ and *ple*^[CC]^ CopyCatcher elements were identified and followed by genomic insertion confirmation by flanking PCR^19^.

### *Drosophila* stocks and genetics

Fly stocks were raised on standard *Drosophila* food under 18 °C with a 12/12 h day/night cycle and experimental flies were raised at 25 °C. The *y*^[ATG-,CC]^, *w*^[ATG-,CC]^ and *ple*^[ATG-,CC]^ CopyCatcher flies with 5′ out-of-frame allele were created either by recombination with existing alleles (*y*^[ATG-,CC]^ with *y*^*[1]*^) or injecting gRNAs expressing by *pCFD3* vector targeting to the site near ATG translational initiation codons (Addgene 49410) into CopyCatcher flies (*w* ^[ATG-,CC]^ and *ple*^[ATG-,CC]^). Compound out-frame CopyCatcher alleles recovered among F_1_ generation progeny were used as the donor chromosome in Fig. 1a. The *y* ^[ATG-,CC]^ and *w*^[ATG-,CC]^ lines were isogenized and made into homozygous stocks, while *ple*^[ATG-,CC]^ remained balanced with TM6 due to the lethality of null mutations in this locus. The distance between ATG^-^ and gRNA cutting sites were 2594 bp, 1694 bp, and 958 bp for *y*^[ATG-,CC]^, *w*^[ATG-,CC]^, and *ple*^[ATG-,CC]^ respectively. These distances are sufficient to prevent detectable co-transmission of ATG^-^ alleles with CopyCatcher elements together from donor to receiver chromosome as a consequence of being captured by HDR-mediated localized gene conversion, which typically extends only 150–200 bp (maximum 1 kb) on either side of the DSB that is being repaired^33,34,93^. The *y*^[ATG-,CC]^, *w*^[ATG-,CC]^ and *ple*^[ATG-,CC]^ flies carrying CopyCatcher elements and ATG^-^ allele were combined in various configurations described in the text with *actin*-Cas9, *vasa*-Cas9, or *nanos*-Cas9 lines on the X chromosome (kindly provided by Valentino M Gantz)^14^, or *vasa*-Cas9 on the third chromosome (BDSC 51324). Fly stocks used for RNAi-based genetic screening were ordered from the Bloomington *Drosophila* stock center. Flies were anesthetized and selected by phenotyping under a Zeiss Stemi 2000 fluorescence microscope for somatic and germline mediated gene-drive experiments, and imaging of compound eyes and thorax. Helicon Focus (v7.6.1 Pro) was used to stack all images. Fiji (OS version) and Photoshop (v20.0.7) were used for adjusting contrast. Microsoft Excel 2019 (v16.30) was used for data collection and GraphPad Prism 8 (v8.2.1) was used for data analysis and display.

### Genomic DNA preparation

Single adult flies were used for genomic DNA preparation according to the manufacturer instructions of Qiagen DNeasy Blood and Tissue Kit^94^. Briefly, flies were crushed using 49 ﻿μl lysis buffer ﻿with 1 mM EDTA, 10 mM Tris pH 8.2 and 25 mM NaCl, and added with 1 ﻿μl Proteinase K to a final concentration of 0.3 mg/ml after the homogenization. Then the reaction mixture was incubated for 37 °C for 30 min, and 95 °C for 2 min. Samples were diluted with 150 μl ddH_2_O and stored in −20 °C.

### Screening for genetic modifiers of CopyCatcher activity

A three-step crossing scheme was employed to screen for genes biasing the frequency of CopyCatcher induced SGC events. Briefly, we recombined the UAS-Cas9 (BDSC 54595) and *ple*^[ATG-,CC]^ on the third chromosome to generate *ple*^[ATG-,CC]^-UAS-Cas9/TM6 firstly, and created an MS1096-GAL4; CyO/Sco or MS1096-GAL4; TM6/Sb stock. We then combined Gal4 and RNAi lines (MS1096-GAL4; UAS-RNAi, as well as targeted mutation lines or UAS over-expressing lines)^51^ according to the insertion site of the shRNA expressing cassettes. Finally, the *ple*^[ATG-,CC]^-UAS-Cas9/TM6 virgins were crossed with males carrying MS1096-GAL4 and RNAi cassette, and all-female progeny were used to scoring SGC.

### Generation of HEK293T cell-based screening system for quantitation of NHEJ and HDR events

We generated a fluorescent-based reporter system in human cells by first inserting a *P2A*-*copGFP* (HEK293T *GAPDH-copGFP* cell line, purchased from ATCC) sequence just before the stop codon of *GAPDH* and isolated a clone carrying one copy insertion by FACS. In addition, a point mutation was generated by NHEJ at the homologous chromosome at the same locus of gRNA targeted, creating a new gRNA targeting site and we termed it as NHEJ allele. We used this cell line for assessing two kinds of somatic HDR events: (1) Plasmid-templated HDR efficiency, by transfection plasmid expressing gRNA targeting to the *copGFP* (gRNA^copGFP^: 5′–CTTCCTCTTGTGCTCTTGCTGGG–3′) as well as a donor DNA plasmid containing promoter-less *mCherry* sequence flanking the *copGFP* cut site. HDR efficiency was scored by the fraction of cells which are GFP^-^mCherry^+^; and (2) Homologous chromosome templated somatic HDR, by transfection gRNA expression plasmid targeting to (gRNA^NHEJ^: 5′–GCCCCAGCAAGAGCACCAAGAGG–3′) the NHEJ allele at the *GAPDH* locus. In this situation, HDR frequency was calculated by single or double copy expression of *GFP*. We evaluated the effect of candidate HDR modulators, which we screened in *Drosophila* in vivo, in altering the efficiency of HDR, first, by transfecting monoallelic *copGFP* expressing cells with guide RNAs targeting each candidate along with spCas9 and confirmed the mutation at the specified locus by Sanger sequencing and consequent reduction in mRNA by qRT-PCR. 48-h after the first transfection, monoallelic *copGFP* cells were subjected to second round of transfection with gRNA^copGFP^ and donor DNA plasmid or gRNA^NHEJ^ only. Samples were harvested at 72 h after transfection, washed with PBS, diluted in FACS buffer (2% FBS, 2 mM EDTA, and 2 mM NaN_3_ in PBS), and sent for FACS analysis with FlowJo 10 (Tree Star, v10.7). Snapgene (v5.0.7) was used for Sanger sequencing analysis. All the guide RNA sequences are listed in the Supplementary Table 1.

### Amplicon-based deep sequencing

Twenty flies of indicated genotype were collected for genomic DNA extraction. Genomic loci spanning the gRNA targets were PCR amplified with gene-specific primers (Supplementary Table 2) added with 5′ tails complementary to the Trueseq adaptors (5′–ACACTCTTTCCCTACACGACGCTCTTCCGATCT–3′ adaptors for forward primers and 5′–GACTGGAGTTCAGACGTGTGCTCTTCCGATCT–3′ for reverse primers). Next, 5 μl of the amplification products were used for the secondary PCR with index-containing primers. The individual PCR products from different crosses were pooled together at the concentration of 10 nM for each sample and subjected to Illumina paired-end 100 bp Amplicon-based deep sequencing. All reads were analyzed with Bowite2 (v2.4.1), and wild-type *white* and *pale* sequences were used as references.

### Single cell colony

We verified our ability to distinguish cells heterozygous versus homozygous for the *GADH-GFP* insertion element in cell populations separated by FACS. We sorted single cells into individual wells of 96-well plates and grew them for ~2 weeks without changing medium until colonies could easily be observed. A set of primers targeting sequences flanking the insertion cassette were used to PCR amplify either heterozygous or homozygous *copGFP*. Two bands were amplified from the heterozygous *GAPDH-copGFP* cell line, one at 1025 bp (*copGFP* allele) and another at 265 bp (NHEJ allele). These heterozygous cells were used as a negative control for copying of the *GAPDH-GFP* element. A homozygous *GAPDH-copGFP* line which we also derived was bi-allelic for *copGFP* (1025 bp) was used as the positive control for SGC in cells initially in a heterozygous condition. We further validated the homozygosity of the single cell colonies by *copGFP*-specific amplification, and then definitively genotyped heterozygous versus homozygous *GAPDH-copGFP* cell lines using a single nucleotide polymorphism that distinguishes the *copGFP* and NHEJ alleles.

### Statistical analysis

The statistical analysis was performed with GraphPad Prism 8 by two-tailed *t*-test or one-way ANOVA. Error bars in figures centered around the mean represent the standard deviation (±SD), and *p*-values (e.g., *p* < 0.001) were used to affirm significance.

### Reporting summary

Further information on research design is available in the Nature Research Reporting Summary linked to this article.

## Supplementary information

Reporting Summary

Supplementary Information

## Data Availability

The sequences of all plasmids used in this study has been deposited into GenBank Database with the accession number as following: yellow CopyCatcher donor plasmid (MW770349), white CopyCatcher donor plasmid (MW770350), ple CopyCatcher donor plasmid (MW770351), mCherry donor plasmid (MW770352). NGS raw data was deposited into GenBank Database with the accession number: white CopyCatcher (SAMN18541175 and SAMN18541176), ple CopyCatcher (SAMN11541177 and SAMN11541178). Source data is provided in this paper as a Source Data File. Other relevant data are available from the authors. Source data are provided with this paper.

## Source data

Source Data
