# Supplementary material for: CopyCatchers are versatile active genetic elements that detect and quantify inter-homolog somatic gene conversion
Source: Nat Commun. 2021 May 11;12:2625. doi: 10.1038/s41467-021-22927-1 (PMC8113449; doi:10.1038/s41467-021-22927-1)
Supplement: Supplementary file 1 — Reporting Summary [file 41467_2021_22927_MOESM1_ESM.pdf]

Corresponding author(s): Ethan Bier
Last updated by author(s): Feb 16, 2021

Reporting Summary

Nature Research wishes to improve the reproducibility of the work that we publish. This form provides structure for consistency and transparency in reporting. For further information on Nature Research policies, see our Editorial Policies and the Editorial Policy Checklist.

Statistics

For all statistical analyses, confirm that the following items are present in the figure legend, table legend, main text, or Methods section.

n/a	Confirmed	
☐	☒	The exact sample size (n) for each experimental group/condition, given as a discrete number and unit of measurement
☐	☒	A statement on whether measurements were taken from distinct samples or whether the same sample was measured repeatedly
☐	☒	The statistical test(s) used AND whether they are one- or two-sided<br>*Only common tests should be described solely by name; describe more complex techniques in the Methods section.*
☒	☐	A description of all covariates tested
☒	☐	A description of any assumptions or corrections, such as tests of normality and adjustment for multiple comparisons
☐	☒	A full description of the statistical parameters including central tendency (e.g. means) or other basic estimates (e.g. regression coefficient) AND variation (e.g. standard deviation) or associated estimates of uncertainty (e.g. confidence intervals)
☐	☒	For null hypothesis testing, the test statistic (e.g. F, t, r) with confidence intervals, effect sizes, degrees of freedom and P value noted<br>*Give P values as exact values whenever suitable.*
☒	☐	For Bayesian analysis, information on the choice of priors and Markov chain Monte Carlo settings
☒	☐	For hierarchical and complex designs, identification of the appropriate level for tests and full reporting of outcomes
☒	☐	Estimates of effect sizes (e.g. Cohen's d, Pearson's r), indicating how they were calculated

Our web collection on statistics for biologists contains articles on many of the points above.

Software and code

Policy information about availability of computer code

Data collection	Zeiss Stemi 2000 fluorescence microscope were used to acquire images, Microsoft Excel 2019 (v16.30) were used for data collection.
Data analysis	Fiji (OS version) and Photoshop (Photoshop CC v20.0.7) were used to contrast, Helicon Focus (v7.6.1 Pro) was used to stack all images. FACS data was analyzed using FlowJo 10 (Tree Star, v10.7). GraphPad Prism 8 (v8.2.1) was used for data analysis and display.Bowite2(v2.4.0) was used for NGS sequence analysis. SnapGene (v5.0.7) was used for Sanger sequencing analysis.

For manuscripts utilizing custom algorithms or software that are central to the research but not yet described in published literature, software must be made available to editors and reviewers. We strongly encourage code deposition in a community repository (e.g. GitHub). See the Nature Research guidelines for submitting code & software for further information.

Data

Policy information about availability of data

All manuscripts must include a data availability statement. This statement should provide the following information, where applicable:

- Accession codes, unique identifiers, or web links for publicly available datasets
- A list of figures that have associated raw data
- A description of any restrictions on data availability

The sequences of all plasmids used in this study has been deposited into GenBank Database with the accession number as following: yellow CopyCatcher donor plasmid (MW770349), white CopyCatcher donor plasmid (MW770350), ple CopyCatcher donor plasmid (MW770351), mCherry donor plasmid (MW770352). NGS raw data was deposited into GenBank Database with the accession number: white CopyCatcher (SAMN18541175 and SAMN18541176), ple CopyCatcher (SAMN11541177 and SAMN11541178). Source data is provided in this paper as a Source Data File. Other relevant data are available from the authors.

Field-specific reporting

Please select the one below that is the best fit for your research. If you are not sure, read the appropriate sections before making your selection.

- [x] Life sciences
- [ ] Behavioural & social sciences
- [ ] Ecological, evolutionary & environmental sciences

For a reference copy of the document with all sections, see nature.com/documents/nr-reporting-summary-flat.pdf

Life sciences study design

All studies must disclose on these points even when the disclosure is negative.

Sample size	Our previous experience of similar analysis for gene drive suggested a number size of >5 with single fly pair crosses is usually representative. In each of our experimental condition, we have collected data from at least 6 crosses (Lopez et al., 2020). For experiments involving quantification of somatic SGC with ple CopyCatcher, at least 15 flies were counted. The sample size for quantifying SGC with white CopyCatcher were determined by the number of progeny within each single pair cross (at least 20 tested animals were used). For experiments in HEK293T cells, three replicates were conducted and sample size was determined with the number of cells within each replicates (12 wells plates used, n>12000 cells).
Data exclusions	Fly crosses with no progeny due to contamination or other causes were removed from the analysis (Supplementary Fig. 4a). Other data were not excluded from analysis.
Replication	At least 5 independent technical replicated were conducted for gene drive elements inheritance, and plotted as individual data points. For somatic gene conversion, at least 15 flies were used for quantifying SGC with ple CopyCatcher and plotted with individual data points. And the semi-quantification for SGC with white CopyCatcher were conducted with at least 10 single pair crosses. For HEK293T cell line experiments, 3 replicates were conducted. All replicates except one for Supplementary Fig. 4a were successful for all experiments. Other replication details are addressed in the text.
Randomization	F1 progeny were randomly collected from F0 crosses to perform F1 crosses. We also randomly selected the F1 flies for SGC quantification with ple CopyCatcher. It was not applicable for SGC analysis with white CopyCatcher and FACS analysis in HEK293T cell line experiments since all samples were used for analysis.
Blinding	In all fly experiments performed we had checked fluorescence presence in the eyes of fruit flies. The SGC analysis we performed by either counting numbers of pale thoracic bristles or existence of white patches in the compound eyes of flies. Investigators were not blinded with all these scoring types. Investigators were not blinded with HEK293T cell line experiments since fluorescence presence was used for cell sorting.

Reporting for specific materials, systems and methods

We require information from authors about some types of materials, experimental systems and methods used in many studies. Here, indicate whether each material, system or method listed is relevant to your study. If you are not sure if a list item applies to your research, read the appropriate section before selecting a response.

Materials & experimental systems

n/a	Involved in the study	
x		Antibodies
	x	Eukaryotic cell lines
x		Palaeontology and archaeology
	x	Animals and other organisms
x		Human research participants
x		Clinical data
x		Dual use research of concern

Methods

n/a	Involved in the study	
x		ChIP-seq
	x	Flow cytometry
x		MRI-based neuroimaging

Eukaryotic cell lines

Policy information about cell lines

Cell line source(s)	HEK293T cells were purchased from ATCC.
Authentication	None of the cell lines have been authenticated.
Mycoplasma contamination	Cell lines were not tested for mycoplasma contamination but no indication of contamination was observed.
Commonly misidentified lines (See ICLAC register)	No commonly misidentified cell lines were used.

Animals and other organisms

Policy information about studies involving animals; ARRIVE guidelines recommended for reporting animal research

Laboratory animals	Wild-type W118 and Oregon-R flies preblastoderm stage eggs (both female and males) were used for generated the transgenic animals.
Wild animals	No wild animals were used in this study.
Field-collected samples	No field-collected samples were used in this study.
Ethics oversight	Drosophila melonogaster is an invertebrate and is exempt from IACUC oversight.

Note that full information on the approval of the study protocol must also be provided in the manuscript.

Flow Cytometry

Plots

Confirm that:

- [x] The axis labels state the marker and fluorochrome used (e.g. CD4-FITC).
- [x] The axis scales are clearly visible. Include numbers along axes only for bottom left plot of group (a 'group' is an analysis of identical markers).
- [x] All plots are contour plots with outliers or pseudocolor plots.
- [x] A numerical value for number of cells or percentage (with statistics) is provided.

Methodology

Sample preparation	Samples were harvested at 72 hours after transfection, washed with PBS, diluted in FACS buffer (2% FBS, 2mM EDTA and 2 mM NaN3 in PBS) and sent for FACS analysis.
Instrument	FACS experiments were performed with BD Fortessa.
Software	FlowJo 10 was used for data analyzed.
Cell population abundance	A minimum of 12,000 cells / sample were acquired for flow cytometry.
Gating strategy	FSC-A/SSC-A (cell of interest)>FSC-W/FSC-H (doublet exclusion). Live cells were analyzed according to Fluorescence expression.

- [x] Tick this box to confirm that a figure exemplifying the gating strategy is provided in the Supplementary Information.
