# Supplementary material for: CopyCatchers are versatile active genetic elements that detect and quantify inter-homolog somatic gene conversion
Source: Nat Commun. 2021 May 11;12:2625. doi: 10.1038/s41467-021-22927-1 (PMC8113449; doi:10.1038/s41467-021-22927-1)
Supplement: Supplementary file 2 — Supplementary Information [file 41467_2021_22927_MOESM2_ESM.docx]

**­­­Supplementary Information**

**­­CopyCatchers are versatile active genetic elements that detect and quantify interhomolog somatic gene conversion**

Zhiqian Li^1^, Nimi Marcel^2,3^, Sushil Devkota^1^, Ankush Auradkar^1^, Stephen M. Hedrick^2,3^,

Valentino M Gantz^1^, Ethan Bier^1,4*^

^1^ Section of Cell and Developmental Biology, University of California San Diego, La Jolla, CA 92093, USA.

^2^ Section of Molecular Biology, University of California San Diego, La Jolla, CA 92093, USA.

^3^ Department of Cellular and Molecular Medicine, University of California San Diego, La Jolla,

CA 92093, USA.

^﻿4^ Tata Institute for Genetics and Society-UCSD, La Jolla, CA 92093-0335, USA.

* Correspondence: Ethan Bier, email: ebier@ucsd.edu

**Supplementary Note 1. Maternal inherited Cas9 contributes to SGC positively.**

Given the maternal inherited Cas9 sustained drive in germline cells (Guichard et al., 2019)^1^, we tested whether shadow drive could also be observed in both somatic and germline cells with CopyCatchers elements (Supplementary Fig. 3b,c, Supplementary Fig. 4c,d).

Remarkably, in all crosses (maternal pedigrees = M-M Cas9, or paternal pedigrees = P-M Cas9) with *w*^[ATG-,CC]^ CopyCatcher and *actin*-Cas9 or *vasa*-Cas9 sources, F_2_ individuals exhibited comparable levels of SGC whether or not they expressed Cas9 zygotically (i.e., carried a genetic source of Cas9, Supplementary Fig. 3b). We note that using the germline-specific *nanos*-Cas9 source inherited from grandmother, maternal only inherited Cas9 (M-M: ZG♂+MC♀) generated 24% of individuals with double-sided SGC, while maternal + zygotic inherited Cas9 (M-M: ZG♂+ZMC♀) increased more than two-fold (54%) in F_2_ *w*^[ATG-,CC]^/*nanos*-Cas9 females (Supplementary Fig. 3b), indicating roughly equal contributions of maternal and zygotic Cas9 expression for this driver. A similar SGC frequency trend was observed with Cas9 inherited from grandfather (P-M Cas9), with notably less of a difference in SGC frequencies between maternal-only versus maternal plus zygotic *nanos*-Cas9 producing mothers, presumably reflecting differential effects of one versus two generations of additive maternal Cas9 deposition. These results suggested specific maternal Cas9 levels could sustain maximal SGC.

A parallel experiment using *ple*^[ATG-,CC]^ CopyCatcher was conducted by quantifying SGC frequency with the fraction of pale thoracic bristles (Supplementary Fig. 3c). F_2_ female Cas9/+; *ple*^[ATG-,CC]^/+ progeny sustained 39%, 48% and 46% SGC for maternal + zygotic provision of Cas9 (ZG♂+ZMC♀) with the *actin*, *vasa* and *nanos*-Cas9 sources respectively. Similarly, maternal-only deposition of Cas9 (+; *ple*^[ATG-,CC]^ /+) resulted in 39%, 43%, 34% of SGC with the same Cas9 drivers (Supplementary Fig. 3c). And the results from F_2_ males were nearly identical to those of F_2_ females (Supplementary Fig. 3c). Cumulatively, these results reveal that maternally provided Cas9-gRNA complexes not only contribute significantly to SGC, but also are sufficient to sustain it at high levels on their own.

We compared the germline gene conversion (GGC) efficiencies in progeny when F_2_ Cas9^+^ (*w*^[ATG-,CC]^/Cas9 or Cas9/+; *ple*^[ATG-,CC]^/+) versus F_2_ Cas9^-^ (*w*^[ATG-,CC]^/+ and +; *ple*^[ATG-,CC]^/+) were crossed to w^–^ counterparts. We observed that CopyCatcher transmission to F_3_ progeny from F_2_ *w*^[ATG-,CC]^ and *ple*^[ATG-,CC]^ flies was comparable for both Cas9^+^ (ZG♂+ZMC♀) and Cas9^–^ (ZG♂+MC♀), with transmission frequencies in the 80-97% range (Supplementary Fig. 4c,d). These results reinforce the conclusion that maternally deposited Cas9 alone is sufficient to produce nearly maximal CopyCatcher drive activity as this prominent maternal effect applies both to somatic cells and the germline. Collectively, all these results confirmed both spatial and temporal distribution of Cas9 contribute significantly to SGC efficiency.

**Supplementary Note 2. Co-inheritance of CopyCatcher and Cas9 affect both SGC and GGC negatively.**

The negative effects of maternal deposited Cas9/gRNA complex have been manifested by higher NHEJ generated during the early embryogenesis, which precluded the later HDR-mediated repair in germline cells. We tested this hypothesis by crossing co-inheriting CopyCatcher elements with Cas9 together from either paternally or maternally, which mimics the “full-drive” active genetic elements and we refer to as the ZMGC crossing scheme (Zygotic plus Maternal inherited both CopyCatcher/gRNA and Cas9) or ZGC (Zygotic inherited both CopyCatcher/gRNA and Cas9) configuration (Supplementary Fig. 3d). ZGC crosses produced an average of 85% SGC rates and ZMGC crosses generated only 25% in F_1_ *y*^+^,*w*^[ATG-,CC]^/y^[^*^vasa^*^-Cas9]^,*w*^+^ female flies (Supplementary Fig. 3e). Similarly, dual inheritance of the *ple*^[ATG-,CC]^ and Cas9 from F_0_ fathers resulted in an average SGC frequencies of 41% (assessed by percentage of pale thoracic bristles) but only 15% when inherited from F_0_ mothers (Supplementary Fig. 3f).

A similar reduction in GGC drive events was observed when F_1_ mothers inherited the *w*^[ATG-,CC]^ and *vasa*-Cas9 transgenes from their F_0_ mothers (57% for F_2_ females, 53% for F_2_ males) compared to their F_0_ fathers (80% for both F_2_ females and males) scored by transmission of CFP to F_2_ progeny (Supplementary Fig. 4e). The same skew in F_1_ GGC drive was observed using F_0_ maternally versus paternally inherited *ple*^[ATG-,CC]^ CopyCatcher elements plus *vasa*-Cas9 elements (Supplementary Fig. 4f). Here we only scored the GGC efficiencies for F_1_ mothers inheriting both *ple*^[ATG-,CC]^ CopyCatcher element and *vasa*-Cas9 either from F_0_ grandmothers versus to F_0_ grandfathers to their F_2_ progeny (Supplementary Fig. 4f). Again, the paternal crosses sustained higher GGC rates (97% for F_2_ females and 95% for F_2_ males) than the maternal crosses (42% for F_2_ females and 41% for F_2_ males).

**Supplementary Note 3. Distinct NHEJ allele distribution in co-inheritance crosses.**

We also performed a amplicon-based deep sequencing analysis for F_1_ *y*^+^,*w*^[ATG-,CC]^/*y*^[^*^vasa^*^-Cas9]^,*w*^+^ and *y*^[^*^vasa^*^-Cas9]^/+; *ple*^[ATG-,CC]^/+ females to investigate the composition of indels generate by NHEJ repair in somatic cells with ZMGC and ZGC crossing (Supplementary Fig. 5). More than 60,000 raw reads were produced for each group. In according to the editing frequency at each nucleotides, we identified the distance at each side between gRNA targets to the nearest exon in F_1_ *y*^+^,*w*^[ATG-,CC]^/*y*^[^*^vasa^*^-Cas9]^,*w*^+^ flies, and it was 973 nucleotides from both ZMGC and ZGC crossing at the left side, and 1243 or 731 nucleotides at the right side in ZMGC and ZGC crossing respectively (Supplementary Fig. 5a,b). This data confirmed all NHEJ created by *w*^[ATG-,CC]^ would be within the safe window and not affecting expression of DsRed and homozygous mutant phenotype. In parallel, we found the distance in *y*^[^*^vasa^*^-Cas9]^/+; *ple*^[ATG-,CC]^/+ females were 915 nt and 928 nt at the left side with ZMGC and ZGC crossing respectively, and 17 nt and 1 nt at the right side (Supplementary Fig. 5e,f). The narrow safe window between gRNA targeting site and exon 2 of *ple* gene was caused by its shorter intron 1, which is 984 bp. Since the editing frequency at the edge sites was very low, which was less than 1 percent out from the total un-copying alleles that were mutated through NHEJ repair, as equal to less than 0.5% out from the total alleles once taking the copying events in account (Supplementary Fig. 5e,f), we confirmed the NHEJ alleles created by *ple*^[ATG-,CC]^ were phenotypically mutated mostly, which in turn further proved CopyCatcher could track SGC efficiently and accurately.

With deep sequence data, we did a further analysis for the abundance of each NHEJ allele. We characterized 866 types of alleles for ZMGC and 1228 alleles for ZGC crossing in F_1_ *y*^+^,*w*^[ATG-,CC]^/*y*^[^*^vasa^*^-Cas9]^,*w*^+^ flies (Supplementary Fig. 5c,d). A similar phenomena was observed for F_1_ *y*^[^*^vasa^*^-Cas9]^/+; *ple*^[ATG-,CC]^/+ females, with 448 different types of alleles identified from ZMGC crossing while 1767 alleles from ZGC crossing (Supplementary Fig. 5g,h). In addition, both F_1_ females from ZMGC crossing generated several dominant alleles with significantly higher fraction according to the allele reads distribution, while alleles from ZGC crossing presented more equally to each other (Supplementary Fig. 5c,d,g,h). These results further support our hypothesis that high levels of maternally provided Cas9-gRNA complexes result in early NHEJ events, which is transmitted into descendent cells after rounds of cell division and accumulates as dominant presence.

**Supplementary Note 4. Summary of temporal and spatial contribution of somatic Cas9 on SGC.**

An important finding from our systematic analysis of different genetic CopyCatcher configurations was that maternally deposited Cas9 stores in the egg could exert either positive or negative effects on SGCs in somatic cells. These various results can be distilled into the following working model. When Cas9 is predominantly expressed in somatic cells during later stages of development (zygotic Cas9, providing Cas9 paternally and denoted as ZGC or ZC), the frequency of SGC improves with increased levels of zygotically expressed Cas9. An opposite effect is observed, however, when high levels of Cas9 are present at early developmental stages, as can result from maternal deposition. We hypothesize that during these earlier stages, HDR mechanisms are not efficient and that DSB are often are repaired by NHEJ leading to indels, which then preclude subsequent HDR-mediated SGC events. Based on these guiding principles, we can infer that the relative levels of somatic Cas9 expression follow the sequence: *actin*-Cas9>*vasa*-Cas9>*nanos*-Cas9, with corresponding increases in SGC frequency observed in paternal crossing schemes wherein fathers carry the source of Cas9. With maternal crossing schemes, however, both maternal deposition and zygotic expression contribute to Cas9 levels which has two effects: increasing early NHEJ and then promoting HDR later in cells where NHEJ did not occur. This effect is most notable for the broadly expressed *actin*-Cas9 and *vasa*-Cas9 sources, leading to reduced SGC frequencies. In the case of *nanos*-Cas9, which is restricted primarily to posterior regions of the embryo that give rise to germ cells, zygotic expression of Cas9 is too low to sustain appreciable SGC. Thus, in this special case, while maternal deposition of Cas9 again results in NHEJ events that limit further HDR events, it also provides sufficient basal levels of Cas9 to promote HDR at later stages resulting in net increased rates of SGC for maternal versus paternal crossing schemes. In combination with analysis of SGC rates when Cas9 is delivered from grandmothers versus grandfathers (the most efficient scenario), we conclude that maternal deposition of Cas9 and gRNA can exert dual opposing effects on both somatic and germline transmission based on the developmental timing of its delivery. These evidences provide us a new insight to optimize the crossing configuration to achieve the highest SGC.


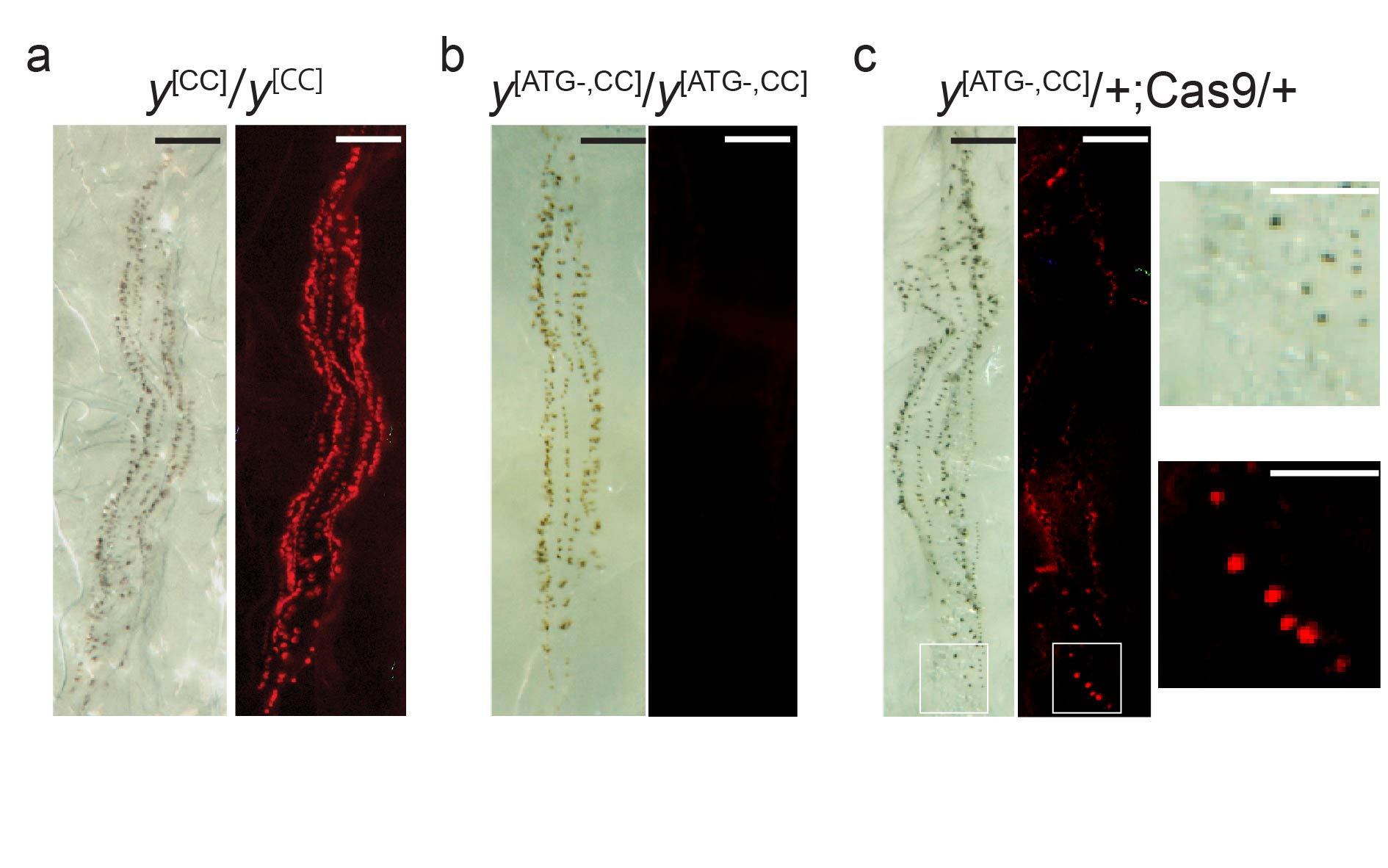
**­­­**

**Supplementary Figure 1**

Mosaic clones of somatic gene conversion (SGC) in *y*^[ATG-,CC]^,*w*^-^/*y*^+^,*w*^-^; Cas9/+ flies.

**a,** The fluorescent CopyCatcher DsRed reporter is expressed in the same pattern as the endogenous *yellow* gene in larval epidermal cells giving rise to ventral denticle hairs. **b**, Loss of fluorescence and yellow pigmentation at ventral denticle belt dots by combining the *y*^[1]^ mutation with *y*^[CC]^. **c**, Mosaic clones in *y*^[ATG-,CC]^,*w*^-^/*y*^+^,*w*^-^;Cas9/+ indicating somatic gene conversion events in larval epidermal cells. A *vasa*-Cas9 placed on the third chromosome was used for crossing. Magnification of the insets labeled with white lines were showed in the right panels. Five biological independent flies were observed. Scale bars stand for 100 pixels in **a**, **b** and left two panels of **c**. Scale bars in the right most panels of **c,** which are the higher magnification view of areas delineated by white boxes in the left, stand for 50 pixels.

**
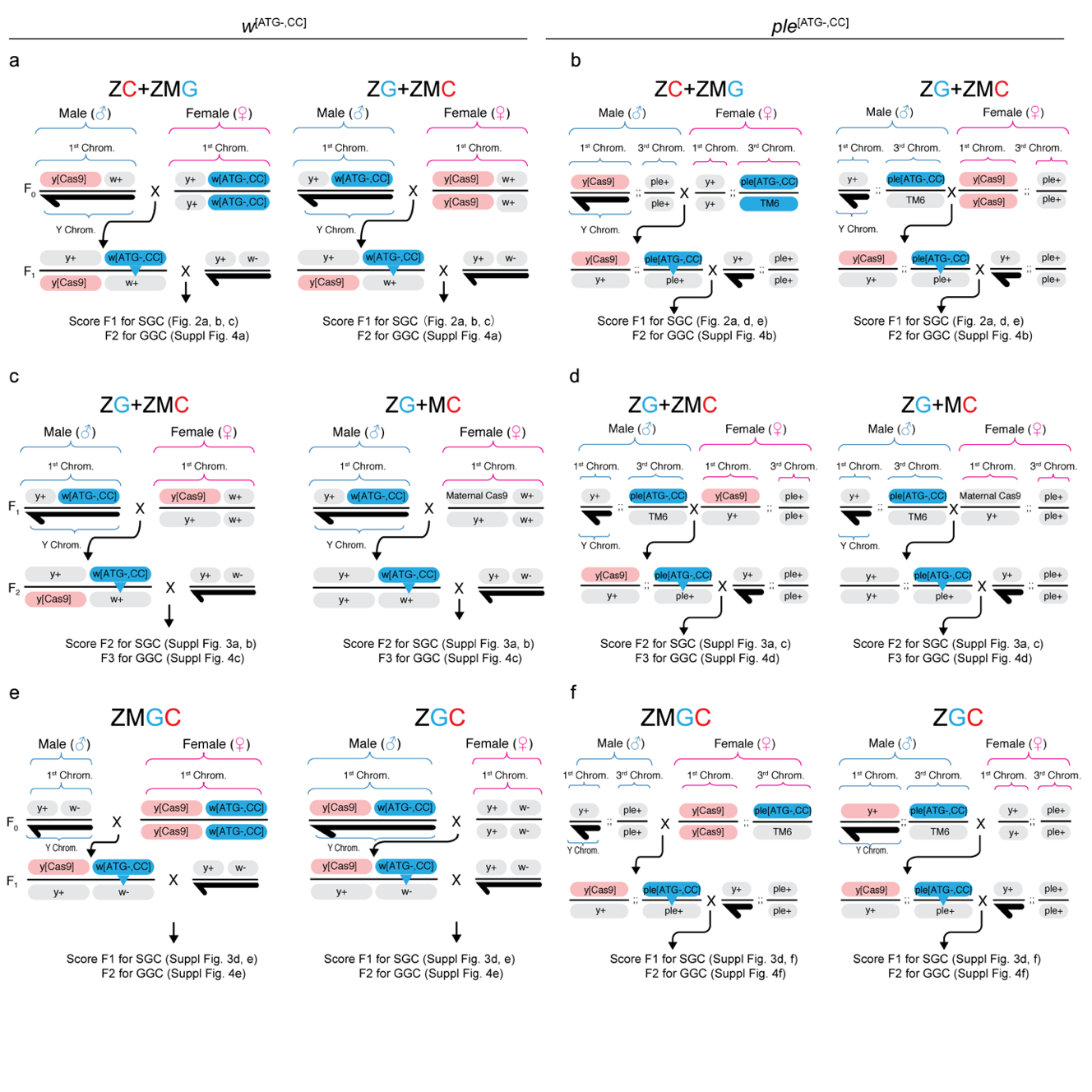
**

**Supplementary Figure 2**

**Cross schemes. a and b,** Cross schemes for providing Cas9 and CopyCatcher separately (ZC: Cas9 inherited from F_0_ male founders, ZMC: Cas9 inherited from F_0_ female founders) for *w*^[ATG-,CC]^ (**a**) and *ple*^[ATG-,CC]^ (**b**). Three Cas9 lines, *actin*-Cas9, *vasa*-Cas9 and *nanos*-Cas9, which all placed at *yellow* loci and marked with *3XP3-DsRed* were used here. **c and d**, Cross schemes for testing the effects of maternal inherited Cas9 protein on SGC. A *vasa*-Cas9 source inserted into the *yellow* locus was crossed to isogenic Oregon-R flies and F_1_ *y*^[Cas9]^,*w*^+^/*y*^+^,*w*^+^ females were crossed with either *w*^[ATG-,CC]^ or *ple*^[ATG-,CC]^. **e and f**, Cross schemes for comparing SGC sustained by paternally inherited Cas9 and gRNA (ZGC) or zygotically + maternally (ZMGC) for the *w*^[ATG-,CC]^ and *ple*^[ATG-,CC]^ CopyCatchers. The same *vasa*-Cas9 transgene used in panels **a** and **b** was combined with either *w*^[ATG-,CC]^ CopyCatcher on the X chromosome, or *ple*^[ATG-,CC]^ CopyCatcher on the third chromosome. The corresponding figures are similarly labeled.

**
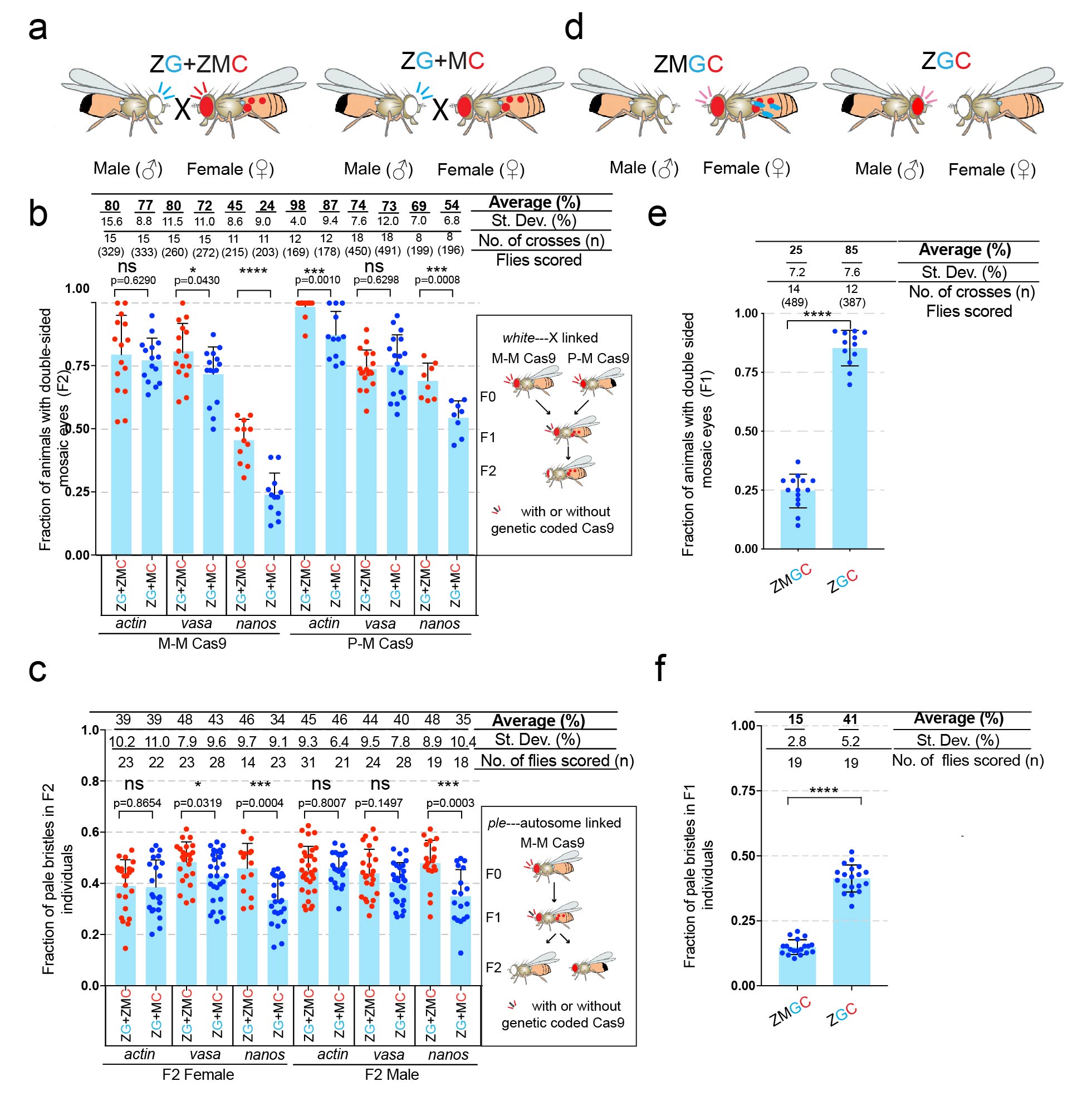
**

**Supplementary Figure 3**

**Maternal deposited Cas9+gRNA complex contributes to SGC both positively and negatively. a,** Cross schemes for detecting SGC with Cas9 inherited maternally plus zygotically (ZG+ZMC) or maternally (ZG+MC). **b and c**, Tabulated SGC efficiency in ZG+ZMC and ZG+MC crossing with *w*^[ATG-,CC]^ (**b**) or *ple*^[ATG-,CC]^ (**c**). SGC outputs were measured by the fraction of F_1_ female progeny having double-sided mosaic eyes (**b**) or the fraction of pale thorax bristles divided by the total number of thorax bristles in individual F_1_ trans-heterozygous females (**c**). **d**, Cross schemes for co-inheriting of both CopyCatcher and Cas9 either maternally (ZMGC) or paternally (ZGC). **e,** **f**, Scoring of SGC efficiency in ZMGC and ZGC crossing with *w*^[ATG-,CC]^ (**e**) or *ple*^[ATG-,CC]^ (**f**). The numbers indicated at the top of the graph represent somatic transmission averages (%), standard deviation, number of cross (represent the number of biological independent crosses) and total number of individual flies counted. Asterisks represent significance assessed by two-tailed *t*-test: four asterisks (*p* < 0.0001), three asterisks (*p < 0.001*), two asterisks (*p < 0.01*), one asterisk (*p < 0.05*), and ns (not significant). Error bars indicate mean ± S.D. Raw data are provided as Source Data file.

**
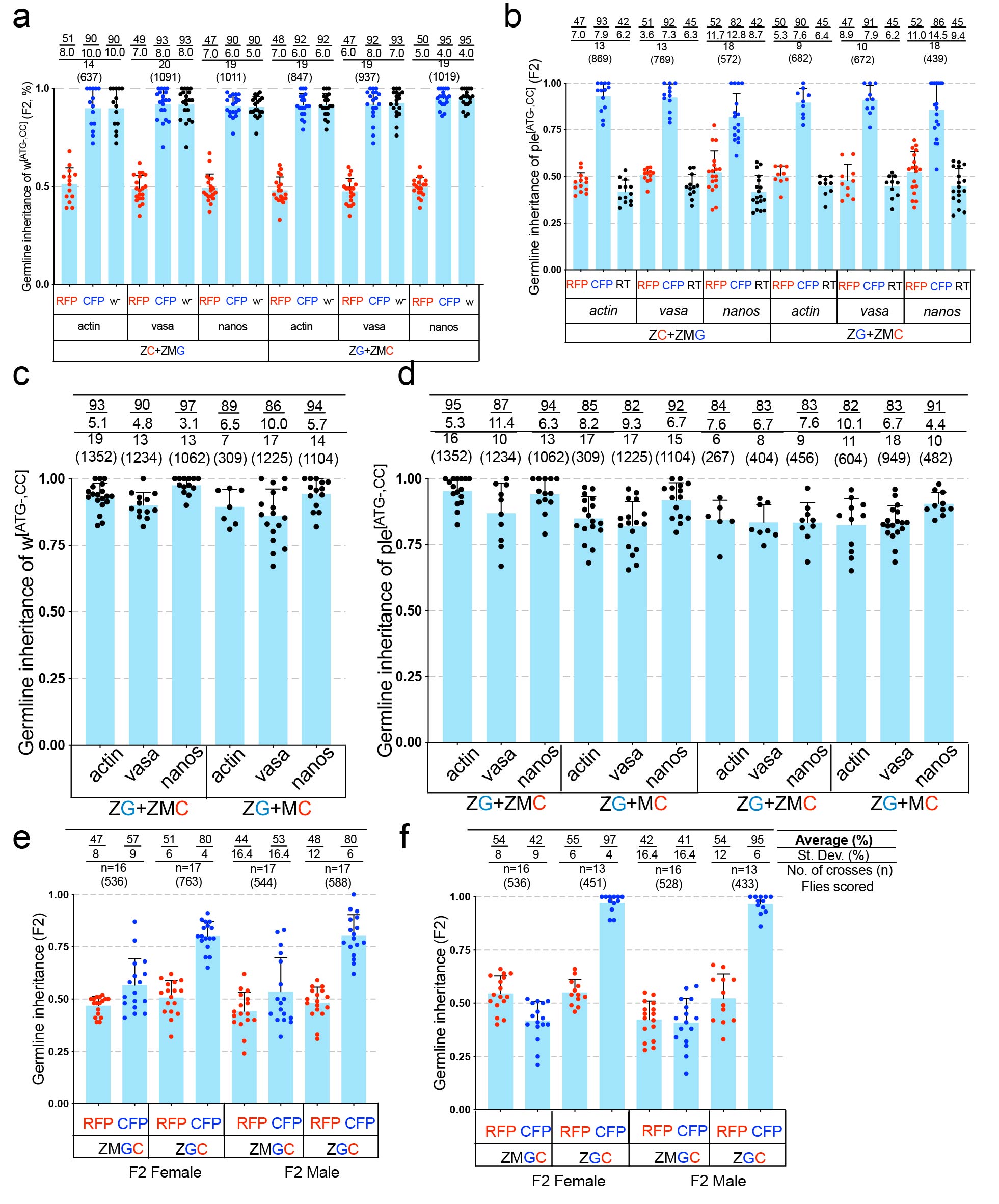
**

**Supplementary Figure 4**

**Germline gene conversion (GGC) efficiency. a and b,** Germline inheritance of *w*^[ATG-,CC]^ (**a**) and *ple*^[ATG-,CC]^ (**b**) in F_2_ progeny from ZC+ZMG or ZG+ZMC. CFP marker is used for GGC quantification, RFP indicates the internal control of Mendelian inheritance, *w*^-^ for the fraction of F_2_ flies with white eyes which marked both donor and receiver chromosome with *w*^[ATG-,CC]^, and RT for the fraction of F_2_ progeny with red thorax bristles that only labeling the receiver chromosome. **c and d,** GGC efficiency in F_3_ progeny with *w*^[ATG-,CC]^ (**c**) and *ple*^[ATG-,CC]^ (**d**) in ZG+ZMC or ZG+MC crosses. **e and f**, GGC with *w*^[ATG-,CC]^ (**e**) and *ple*^[ATG-,CC]^ (**f**) in ZMGC and ZGC crosses. CFP indicates transmission of CopyCatcher in germline cells and RFP serves as an internal control for Mendelian inheritance. Each dot represents single mating. Inheritance average, standard deviation, number of biological independent crosses (n) and total number of scored flies are labeled over each graph.

**
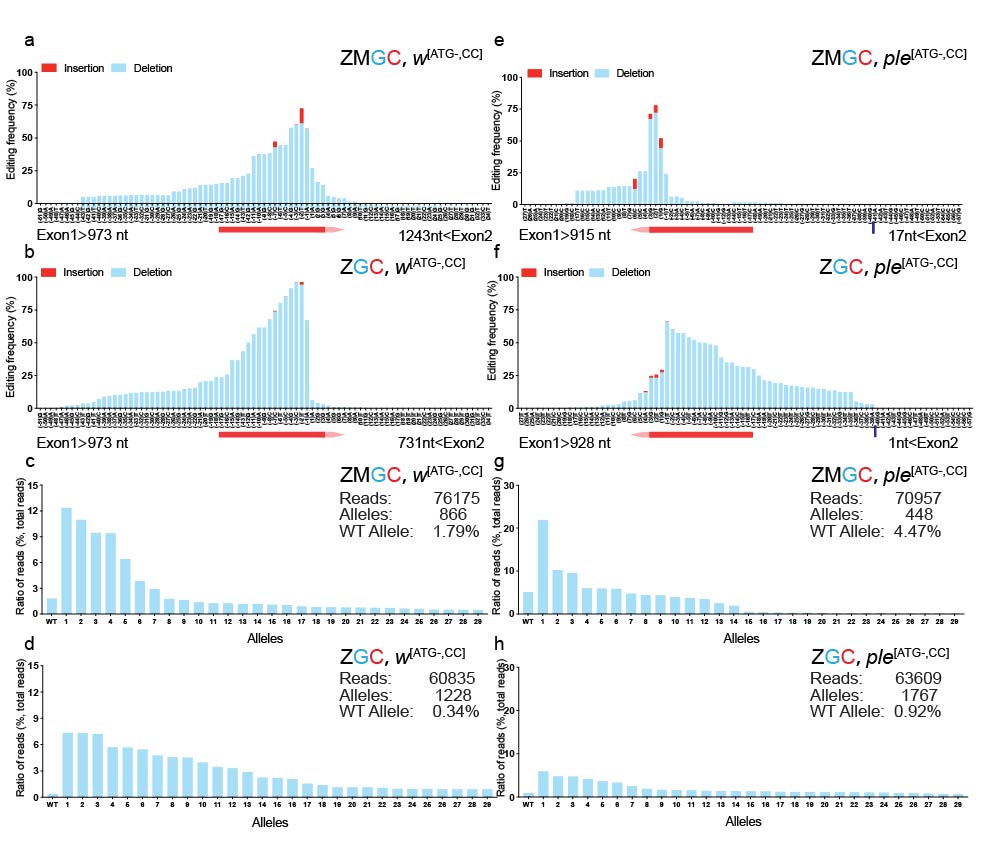
**

**Supplementary Figure 5.**

**Distribution of NHEJ alleles revealed by deep sequencing in somatic cells of ZMGC and ZGC crossing. a and b**, Mutagenesis frequency at each nucleotide site in F_1_ *y*^[Cas9]^,*w*^[ATG-,CC]^/*y*^+^,*w*^+^ females at *w* locus around gRNA targeting site, when Cas9 and gRNA are inherited together from F_0_ females (**a**, ZMGC) or F_0_ males (**b**, ZGC). The frequency was measured by the fraction of sequenced reads mutated at the indicated nucleotides with red bars were insertion while blue bars were deletion. X axis indicated the nucleotides location and Y axis was the editing frequency. gRNA binding sites, distance between the flanking exon and nearest edited nucleotide for each side were listed below X axis. **c and d**, Read abundance for the top 30 alleles. Y axis was frequency of each allele. The total reads, total number of allele types, as well as frequency of wild-type allele were listed as inset in each graph. **e and f**, Mutagenesis frequency at each nucleotide site in F_1_ *y*^[Cas9]^/*y*^+^; *ple*^[ATG-,CC]^/+ females at *ple* loci around gRNA targeting site with ZMG (**e**) and ZGC (**f**) crossing. **g and h**, Distribution of top 30 alleles in flies as in panels **e** and **f**. Raw data are provided as a Source Data file.

**
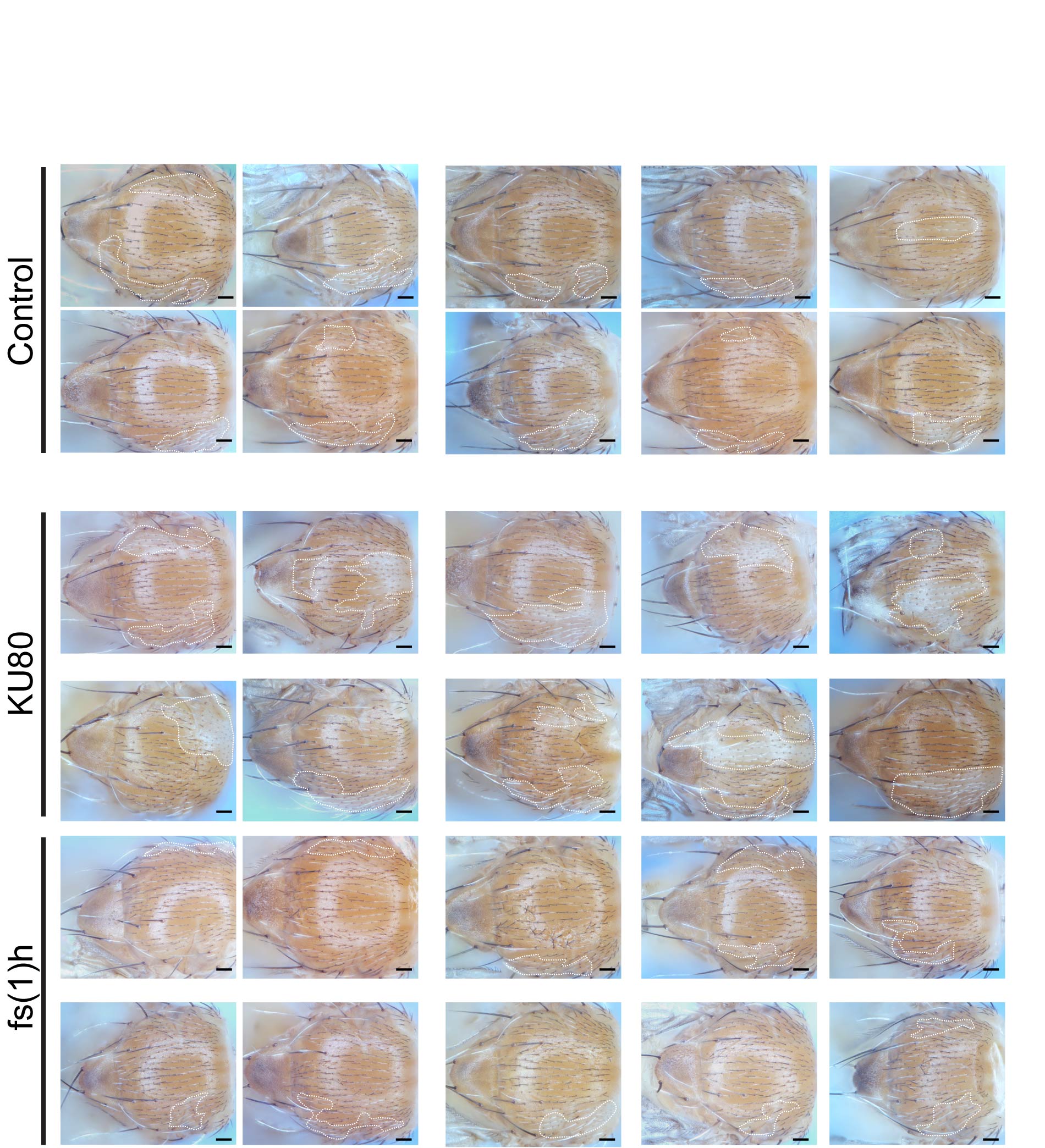
**

**Supplementary Figure 6.**

**Example images with RNAi mediated SGC alteration.** Knocking down *Ku80* increased the fraction of pale bristles while decrease of that was observed with *fs(1)h* down-regulation. For each group, ten biological independent flies were imaged. The dominant patches with pale thoracic epidermal cells and pale bristles were delineated with dotted white lines.

**
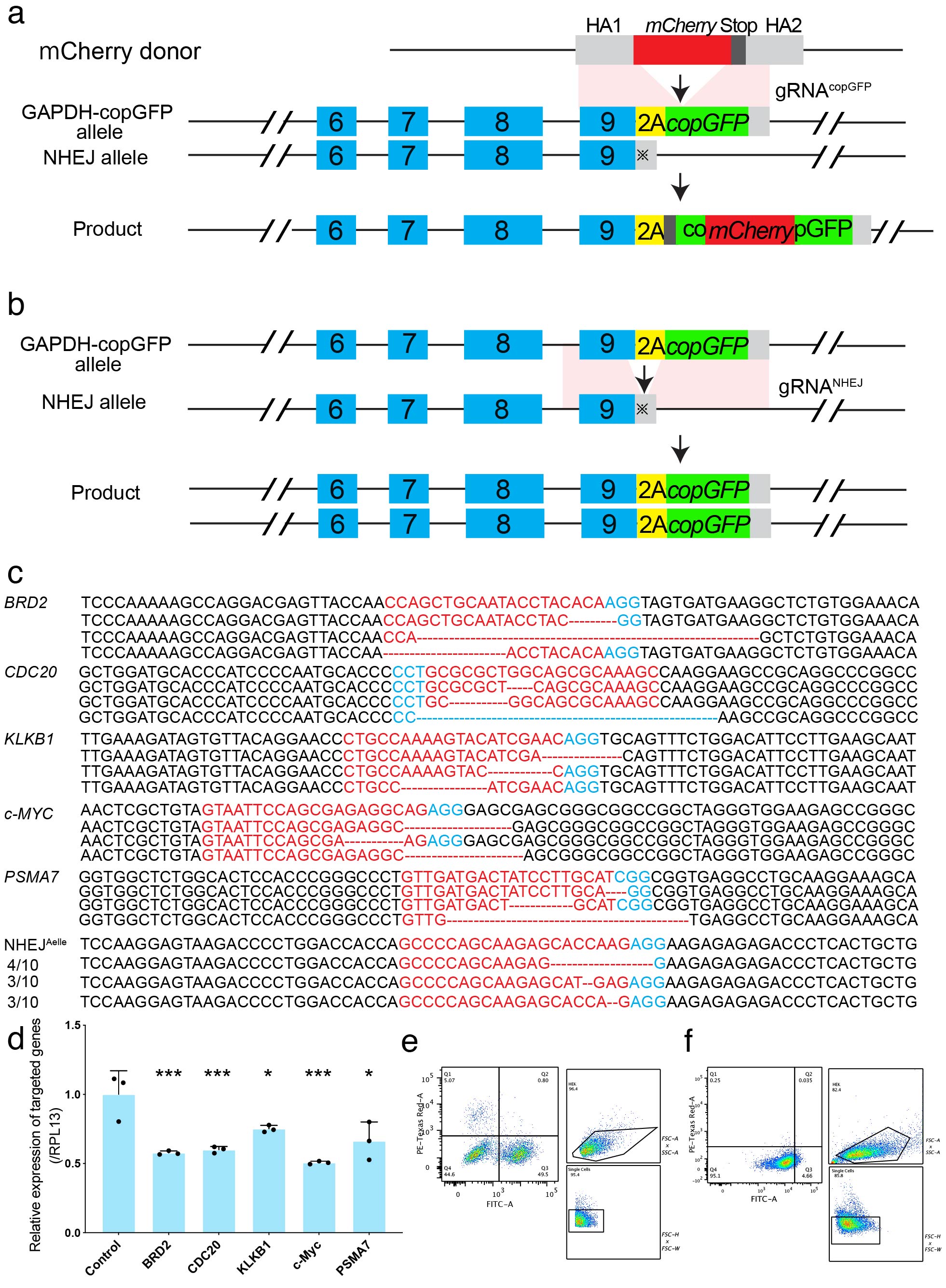
**

**Supplementary Figure 7.**

**Validation of candidate somatic DSB repair modifiers in HEK293T *GAPDH-copGFP* cell line.** **a**, Scheme for exogenous plasmid-templated somatic HDR at *GAPDH-copGFP* loci. A stable cell-line expressing single copy of *P2A-copGFP* is inserted at 3’ terminal region of *GAPDH* (HEK293T *GAPDH-copGFP*). The second allele of endogenous *GAPDH* was a NHEJ indel created by CRISPR/Cas9. Blue arrow indicates gRNA^copGFP^ targeting to 5’ end of *copGFP* which was used for integration of *mCherry*, and donor plasmid contains 1 kb homology arm flanking the gRNA cutting site and a promoter-less *mCherry* coding sequence. **b**, Scheme for homolog chromosome-templated somatic HDR on the NHEJ allele. Black arrow shows gRNA^NHEJ^ targeting to NHEJ allele-specific gRNA. *GAPDH-copGFP* allele was used as DSB repair template and HDR created homozygous *GAPDH-copGFP*. **c**, Indels created by NHEJ at the candidate somatic DSB repair modifiers. Cells after transfection of gRNA targeting each potential somatic HDR promoter or inhibitor were collected at 3 days after transfection and used for genome DNA extraction. Editing results were verified by PCR amplification using primers flanking the gRNA targeting sites and sending for sanger sequence. Red letters indicate gRNA spacers and blue letters are PAM sequences. **d**, Transcriptional level of targeted genes examined by qRT-PCR. *RPL13* was used as the internal control reference. Y axis represents fold change of the relative mRNA expression levels: three biological independent replicates (n) were conducted for each sample. P values were: *BRD2* = 0.0004 (***, one-way ANOVA), *CDC20* = 0.0007 (***, one-way ANOVA), *KLKB1* = 0.0219 (*, one-way ANOVA), *c-Myc* = 0.0001 (***, one-way ANOVA), *PSMA7* = 0.0028 (*, one-way ANOVA). Error bars indicate mean ± S.D. Raw data for **d** is provided as a Source Data file. **e**, **f.** Gating strategy of FACS sorting for plasmid (e) or homolog chromosome-templated (f) experiments.


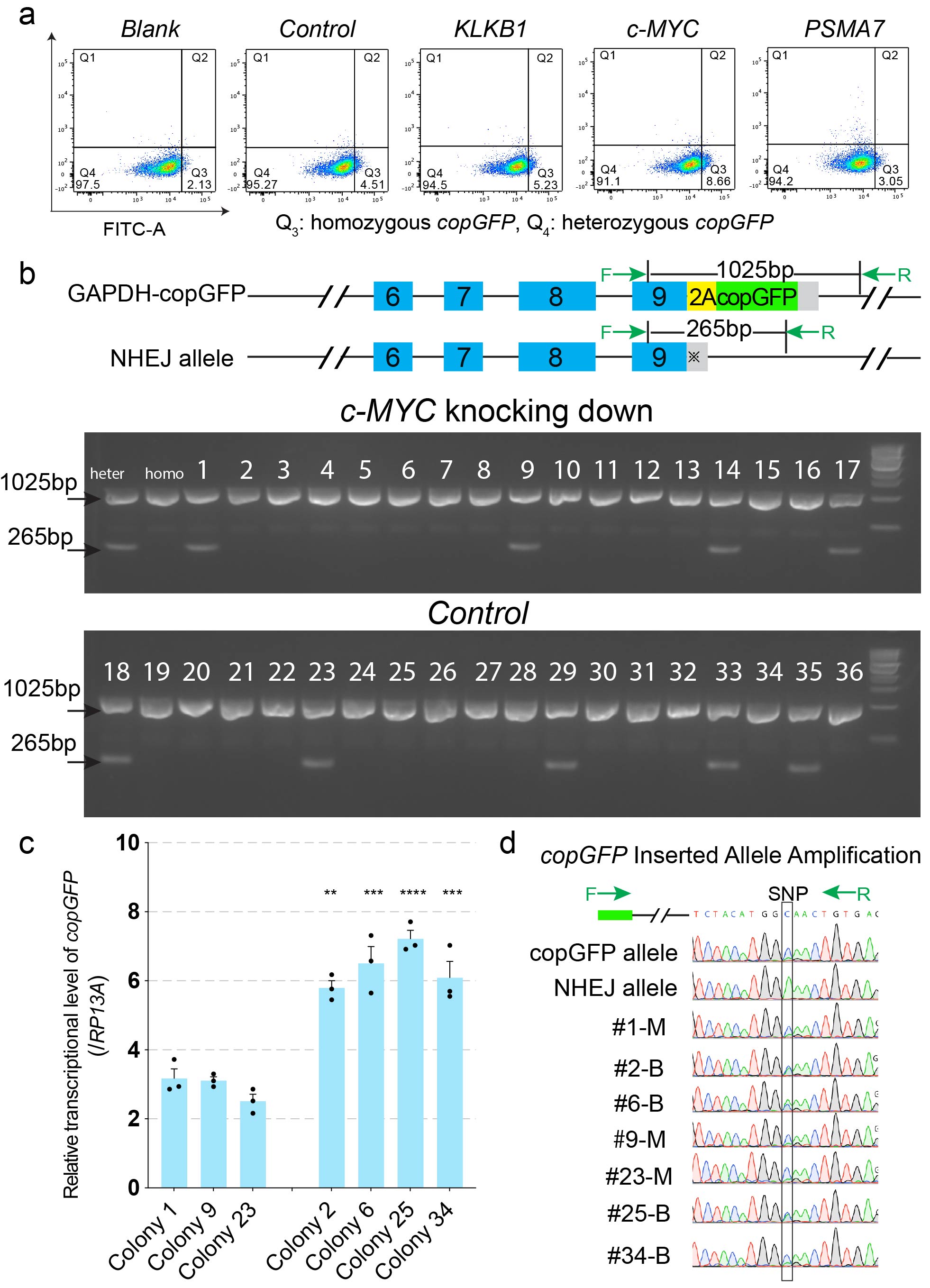


**Supplementary Figure 8.**

**Knocking down *c-MYC* increased homologous chromosome templated HDR recombination (HTR). a,** FACS plots for HTR by GFP intensity with targeted gene knocking down. The section 3 stands for heterozygous *copGFP* and section 4 is homozygous *copGFP*. Blank: transfected with plasmid which only expressing Cas9 at both the first and second transfection. Control: transfected with Cas9 expressing only plasmid at the first transfection, and the secondary transfection with Cas9 and gRNA^NHEJ^ expression on the same plasmid. **b,** PCR validation of the homozygosity for *copGFP* with the single cell colonies. Cells were sorted by FACS and clonal cell lines were established from the population with higher *copGFP* intensity. Primers used for genomic DNA amplification were sited flanking the *copGFP* insertion site. heter: heterozygous *GAPDH-copGFP* negative control, homo: homozygous *GAPDH-copGFP* positive control. Black arrows show the size of bands. NEB 1kb DNA marker was used to indicate fragment length. The bands amplified from NHEJ allele (265 bp) and copGFP allele (1025 bp) were indicated by arrows and length. Seventeen (n) single cell colonies sorted from *c-Myc* knocking down cell population were subjected to PCR amplification, and 19 (n) colonies from control group were performed with amplification using the same primer. **c,** qRT-PCR results for *copGFP* in single cell colonies. P values were: P values were: Colony 2 = 0.0012 (**, one-way ANOVA), Colony 6 = 0.0002 (***, one-way ANOVA), Colony 25 < 0.0001 (****, one-way ANOVA), Colony 34 = 0.0006 (***, one-way ANOVA). Error bars indicate mean ± S.D. Three biological independent replicates (n) were performed. **d,** Distinguishing bi-allelic or mono-allelic *copGFP*. Amplification was performed with a forward primer (F) sited in *copGFP* and reverse primer (R) targeted to the common sequences between the donor and receiver chromosome. A SNP was identified at the site of 94 bp downstream of *copGFP* insertion. Green box stands for *copGFP* coding sequence and black line is genome DNA. B: bi-allelic *copGFP*, M: mono-allelic *copGFP*. SNP was indicated by black rectangle. Sanger sequencing was used for identifying mono-allelic or bi-allelic *GAPDH-copGFP*. Green arrows indicate primers used for amplification. Raw data in **b** and **c** are provided as a Source Data file.

**Supplementary Reference:**

1. Annabel, G. et al. Efficient allelic-drive in *Drosophila*. *Nat Commun*. **10**, 1640 (2019).

**Supplementary Table 1: gRNA sequences used in this study.**

| **Target** | **Sequences (5’-3’)** |
| --- | --- |
| *Dmyellow* | GACTGAAGTACATATAGCCGGGG |
| *Dmwhite* | GCAGTGAAGCCTCGCTAAGGAGG |
| *Dmple* | GAAGTCCGTGTTAGTGACACTGG |
| *HsGAPDH* | CTTCCTCTTGTGCTCTTGCTGGG |
| *copGFP* | GAGATCGAGTGCCGCATCACCGG |
| *HsBRD2* | CCAGCTGCAATACCTACACAAGG |
| *HsCBX1* | AAAAGTTCTCGACCGTCGAGTGG |
| *HsCDC20* | TTCCCTGCCAGACCGTATCCTGG |
| *HsCHRNA2* | GACGTGGTGATTGTGCGCTTTGG |
| *HsKLKB1* | CTGCCAAAAGTACATCGAACAGG |
| *Hsc-Myc* | GTAATTCCAGCGAGAGGCAGAGG |
| *HsORC1* | TGCAGTTTTCGATCCAACAAGGG |
| *HsPSMA7* | GTTGATGACTATCCTTGCATCGG |
| *HsTubb2b* | AGACCAGACAATTTCGTGTTTGG |

**Supplementary Table 2: Primers used in this study (5’-3’).**

| **Name** | **Primer sequence (5’-3’)** |
| --- | --- |
| L30-yellowCCIntrongRNA3HA1F | CCTATAAAAATAGGCGTATCACGAGGCCCTTTCGGCAACAACAGGCTACAGAATA |
| L31-yellowCCIntrongRNA3HA1R | CGACTTCTAGAGTCGACCATCATGATGGACCTATATGTACTTCAGTCATAATTG |
| L32-yellowCCIntrongRNA3SAF | CCATCATGATGGTCGACTCTAGA |
| L33-yellowCCIntrongRNA3RedR | CTAGCTCTAAAACCGGCTATATGTACTTCAGTCGACGTTAAATTGAAAATAGGTCTATA |
| L34-yellowCCIntongRNA3CFPF | GACTGAAGTACATATAGCCGGTTTTAGAGCTAGAAATAGCAAG |
| L35-yellowCCIntongRNA3CFPR | GTTTATGTTAAAAGATCCCCGGTTGACGTAGTGCCCCAACTGGGGTAAC |
| L36-yellowCCIntongRNA3HA2F | CTACGTCAACCGGGGATCTTTTAACATAAAC |
| L37-yellowCCIntongRNA3HA2R | CCGTATTACCGCCTTTGAGTGAGCTGATACCCCCCGTTCATCTAAGGCAAC |
| L53-paleSeqR1 | CCTCGGTGAGACCGTAATctgca |
| L58-pale1HA1F | CCTATAAAAATAGGCGTATCACGAGGCCCTTTCGTTGTTCACCCCAAACGAAAACAAC |
| L59-pale1HA1R | CGACTTCTAGAGTCGACCATCATGATGGACCACTGGCAGGATGTGGAGCGGAAA |
| L60-SAR | GCCGCTACCTCCGCTTCCACCGGAACCTCCCACCTGCGGAAGAGAGATAAATCG |
| L61-SAF | GGAGGTTCCGGTGGAAGCGGAGG |
| L62-pale1DsRedR | GCTCTAAAACGTGTCACTAACACGGACTTCGACGTTAAATTGAAAATAGGTCTATA |
| L63-pale1U6F | CGTCGAAGTCCGTGTTAGTGACACGTTTTAGAGCTAGAAATAGCAAGTTAA |
| L64-U6R | CTTTGGGTATGGACGAACTCTACAAATAGATCTTAGTTTGTATTGTCATGTTTTAATAC |
| L65-mCeruleanF | CTATTTGTAGAGTTCGTCCATACCCAAA |
| L66-mCeruleanR | CGAGCTCGCCCGGGGATCTAATTC |
| L67-pale1HA2F | GAATTAGATCCCCGGGCGAGCTCGCCTAGGTCACTAACACGGACTTCAATCTTTTC |
| L68-pale1HA2R | CCGTATTACCGCCTTTGAGTGAGCTGATACCGCCAGGCAAACGCATTTCGTACTT |
| L69-pale2HA1R | CGACTTCTAGAGTCGACCATCATGATGGACTGGCTTTCTTCGGCTCCACGCTTG |
| L70-pale2DsRedR | GCTCTAAAACGGGTGGCTTTCTTCGGCTCCGACGTTAAATTGAAAATAGGTCTATATA |
| L71-pale2U6F | GTCGGAGCCGAAGAAAGCCACCCGTTTTAGAGCTAGAAATAGCAAGTTAAA |
| L72-pale2U6R | CCTAGGCGAGCTCGCCCGGGGAT |
| L73-pale2HA2F | GAATTAGATCCCCGGGCGAGCTCGCCTAGGCCCTGGCTTGAAGGTCAAGGCAA |
| L74-attpF | CTTTTTAATGTTCGCTTAATGCGTATGCATTTAGACCTACGCCCCCAACTGAG |
| L75-attpR | CACTCCTTTCAAGCGCGTGGGATCGATCGTAGTGCCCCAACTGGGGTAACC |
| L76-OpIE2F | GATCGATCCCACGCGCTTGAAAGG |
| L77-OpIE2R | CTTTGGGTATGGACGAACTCTACAAATAGGTTTATCTGACTAAATCTTAGTTTGTA |
| L78-CFPR | GCGAAAAGATTGAAGTCCGTGTTAGTGACCTAGGCGAGCTCGCCCGGGG |
| L157-whiteHA1F | CCTATAAAAATAGGCGTATCACGAGGCCCTTTCGGGTCATTATTTGGCTAGACGAATG |
| L158-whiteHA1R | CGACTTCTAGAGTCGACCATCATGATGGACTAGCGAGGCTTCACTGCTCATC |
| L159-whiteRedF | CTCTAAAACCCTTAGCGAGGCTTCACTGCGACGTTAAATTGAAAATAGGTCTATAT |
| L160-whiteRedR | CGCAGTGAAGCCTCGCTAAGGGTTTTAGAGCTAGAAATAGCAAGTTAAAA |
| L161-whitegHA2F | CTAATTGAATTAGATCCCCGGGCGAGCTCGAGGAGGAAATGCAACAGTTTTCAGG |
| L162-whitegHA2R | GTATTACCGCCTTTGAGTGAGCTGATACCGGGTCACAGGTCGTTGGGCGAT |
| L163-whiteSeq | CGCCATGTCCCTCTATCTCTTTC |
| L303-HsCHRNA2F | CACCGCTACAACCGCTGGGCGCGCC |
| L304-HsCHRNA2R | AAACGGCGCGCCCAGCGGTTGTAGC |
| L305-HsPSMA7F | CACCGTTGATGACTATCCTTGCAT |
| L306-HsPSMA7R | AAACATGCAAGGATAGTCATCAAC |
| L307-HsMYCF | CACCGAGAGGCAGAGGGAGCGAGC |
| L308-HsMYCR | AAACGCTCGCTCCCTCTGCCTCTC |
| L309-HsORC1F | CACCGTGCAGTTTTCGATCCAACAA |
| L310-HsORC1R | AAACTTGTTGGATCGAAAACTGCAC |
| L311-HsUBE2D3F | CACCGGTTTGAAGGGGTAGTCTGT |
| L312-HsUBE2D3R | AAACACAGACTACCCCTTCAAACC |
| L313-HsCBX1F | CACCGCTCGACCGTCGAGTGGTAAA |
| L314-HsCBX1R | AAACTTTACCACTCGACGGTCGAGC |
| L315-HsTUBB2BF | CACCGCTAACCGAATCCATCGTGCC |
| L316-HsTUBB2BR | AAACGGCACGATGGATTCGGTTAGC |
| L317-HsBRD2F | CACCGCCAGCTGCAATACCTACACA |
| L318-HsBRD2R | AAACTGTGTAGGTATTGCAGCTGGC |
| L319-HsKLKB1F | CACCGCACCTGTTCGATGTACTTT |
| L320-HsKLKB1R | AAACAAAGTACATCGAACAGGTGC |
| L321-HsCDC20F | CACCGCTTTGCGCTGCCAGCGCGC |
| L322-HsCDC20R | AAACGCGCGCTGGCAGCGCAAAGC |
| L480-RPL13A2RT-F | GAGATGACTCCACATGCACTAC |
| L481-RPL13A2RT-R | CATACCTGTTGGGTGACAGATAG |
| L482-BRD2RT-F150 | CGGCTTATGTTCTCCAACTGCTA |
| L483-BRD2RT-R150 | GGCAGTAGAGACTGGTAAAGGC |
| L484-CDC20RT-F157 | CGGAAGACCTGCCGTTACATTC |
| L485-CDC20RT-R157 | CAGAGCTTGCACTCCACAGGTA |
| L486-KLKB1RT-F134 | TGTCGGACCATCTGCACCTATC |
| L487-KLKB1RT-R134 | GGAGTAGAGGAACTTGGTGTGC |
| L488-MYCRT-F | CCTGGTGCTCCATGAGGAGAC |
| L489-MYCRT-R | CAGACTCTGACCTTTTGCCAGG |
| L490-PMSA7RT-F | GTGCTTTGGATGACAACGTCTGC |
| L491-PSMA7RT-R | TAGCGGGTGATGTACTCCACAG |
| L559-GAPDHScanF1 | CCCCCACCCCACCCCTTTCACCATTAG |
| L566-GAPDHScanR1 | GCCCACTGCTCACCTCCTGCTATTTGGC |
| L573-copGFPRTF | AGGACAGCGTGATCTTCACC |
| L574-copGFPRTR | CTTGAAGTGCATGTGGCTGT |
